# Proanthocyanidins as Therapeutic Agents in Inflammation-Related Skin Disorders

**DOI:** 10.3390/ijms262010116

**Published:** 2025-10-17

**Authors:** Aleksandra Prokop, Anna Magiera, Monika Anna Olszewska

**Affiliations:** Department of Pharmacognosy, Faculty of Pharmacy, Medical University of Lodz, Muszynskiego 1, 90-151 Lodz, Poland; aleksandra.prokop@umed.lodz.pl (A.P.); anna.magiera@umed.lodz.pl (A.M.)

**Keywords:** proanthocyanidins, molecular mechanisms, biological activity, skin inflammation, wound healing, psoriasis, atopic dermatitis, anti-inflammatory activity, antioxidant activity, photoprotection

## Abstract

Skin diseases, affecting one-third of the population, are a growing global health problem. The complexity of skin architecture, along with diverse symptomatology and intricate pathogenesis of dermatological disorders, highlights the urgent need for novel therapeutic strategies. Effective treatment of impaired wound healing and chronic skin diseases, including atopic dermatitis and psoriasis, remains challenging. Phytoterapeutics are increasingly investigated for their dermatologic potential, with numerous natural products of established use. Proanthocyanidins (PACs), a subclass of polyphenolic compounds, renowned for their anti-inflammatory and antioxidant properties, are promising candidates for novel solutions. This review article synthesizes the recent 25 years of research on biomolecular mechanisms, pharmacological effects, and phytochemical aspects of PACs, in the context of treating inflammatory-related skin problems. The available data highlight pro-regenerative, pro-angiogenic, antioxidative, and anti-inflammatory effects of PACs in accelerating wound closure. Preclinical data suggest their potent ability to mitigate chronic skin inflammatory disorders, including psoriasis and atopic dermatitis. Moreover, their photoprotective properties translate to the prevention of UV-induced skin inflammation. However, critical knowledge gaps remain regarding clinical verification and structure-activity relationships of PACs as dermatologic agents. Further optimization of topical formulation systems for PACs is also pressingly needed. Bridging traditional phytotherapy with novel discoveries in molecular pharmacology and pharmaceutical technology could help to design innovative PAC-based approaches for treating inflammatory skin diseases and impaired wound healing.

## 1. Introduction

The skin, being the most essential part of the integumentary system, serves as the body’s primary line of defense against external factors, including physical and chemical stressors and pathogens [1,2,3,4]. A healthy condition and optimal integrity of the skin are critical for ensuring it functions as a protective barrier and in preserving the physiological homeostasis of the organism. To meet these challenges, the skin is a metabolically active, self-regenerating, multilayered organ capable of undergoing substantial reorganization in response to various stimuli through the cooperation of three main specialized layers [5,6].

The outermost layer is the epidermis, primarily composed of epithelial cells at various stages of differentiation. Keratinocytes comprise 90–95% of the tissue and are the predominant skin cells [2]. The epidermis’ uppermost part (stratum corneum) is formed by keratin-rich dead keratinocytes and is crucial for maintaining the physical barrier. In contrast, the most active part of the epidermis is the constantly renewing stratum basale [7,8,9]. The epidermis initiates the skin’s innate immune response through complex mechanisms. In the damaged or infected area, the activated keratinocytes recognize pathogens by specific receptors, secrete antimicrobial peptides (AMPs), mediate key signaling pathways, such as the nuclear factor κB (NF-κB) pathway, and release pro-inflammatory cytokines, including interleukins IL-1β, IL-6, and IL-8 [2,7,10].

The second skin layer is the dermis, which is primarily composed of connective tissue, comprising the extracellular matrix (ECM) and fibroblasts. The secretion of collagen and elastin (ECM components) by fibroblasts provides the skin with structural integrity, flexibility, and strength [2]. Like keratinocytes, fibroblasts have immunomodulatory functions; upon stimulation, they release various cytokines and chemokines, including IL-1β, IL-6, IL-8, IL-25, and tumor necrosis factor α (TNF-α) [10]. The presence of blood and lymphatic vessels, and nerve endings, makes the dermis crucial for wound healing, mainly by providing signaling molecules that promote tissue repair and regeneration [2].

The third layer, called hypodermis or subcutaneous layer, is primarily comprising loose connective tissue, adipose tissue, fibrocytes, adipocytes, numerous immune cells, and an extensive network of blood and lymphatic vessels [8,9]. While epidermis and dermis play a crucial role in wound healing, the hypodermis contributes an indirect role, supporting energy storage, adipose homeostasis, as well as healing deep wounds, scars, and preserving the skin’s volume and tension [11].

Harmful stimuli like UV radiation, pollution, and pathogens constantly affect the skin, leading to hypersensitivity, chronic inflammation, and functional disorders. Factors such as metabolic diseases, injuries, genetic issues, and aging can worsen these effects, resulting in common skin diseases like acne, dermatitis, cutaneous infections, immune-mediated inflammatory disorders, chronic wounds, and skin cancers [7,12]. Skin diseases rank as the eighth leading cause of the global disease burden, significantly impacting quality of life and well-being across all age groups worldwide [12,13]. Consequently, treating skin diseases has become increasingly challenging, driving a growing interest in new therapies and cosmetic treatments [14].

Natural products and herbal preparations play a vital role in skin healing. They are primarily used in mild skin disorders and in cosmetics to prevent disease progression and premature aging. Numerous medicinal products, such as *Aloe vera* gel, chamomile (*Matricaria chamomilla*) flower extracts and essential oil, calendula (*Calendula officinalis*) flower extracts, Indian pennywort (*Centella asiatica*) herb extracts, green tea (*Camellia sinensis*) leaf extracts, and witch hazel (*Hamamelis virginiana*) leaf extracts, have been used in skin therapies for centuries [15,16]. In addition, pure compounds, either derived from plants or synthetically, such as ascorbic acid, ferulic acid, gallic acid, retinoids, resveratrol, niacinamide, catechins, caffeine, trehalose, mucopolysaccharides, ceramides, and biomimetic peptides, are widely applied in innovative cosmetics [4]. The active constituents of skincare and skin-healing preparations range from low-molecular-weight volatiles or phenolic acids, to high-molecular-weight polysaccharides or tannins. Among tannins, plant proanthocyanidins (PACs) have recently gained attention for their benefits in skin health.

PACs, also known as condensed tannins, are oligomeric or polymeric flavan-3-ol derivatives found throughout the plant kingdom [17,18]. Numerous in vitro and in vivo studies have provided extensive data on the molecular mechanisms by which PACs can manage various dermatological disorders. Their diverse biological activities establish a solid foundation for effective use in promoting wound healing [19,20,21,22,23,24,25,26,27,28,29,30,31,32], treating skin inflammation, including autoimmune inflammatory diseases [33,34,35,36], and preventing photodermatoses [37,38,39]. Research has shown that PACs-enriched plant extracts and isolated PACs can enhance wound healing [19,20,21,22,23,24,25,26,27,28,29,30,31,32] and alleviate inflammatory responses in the skin by influencing the immune system [33,34,35,36], targeting the regenerative functions of skin cells [19,20,21,22,23,24,25,26,27,28,29,30,31,32], promoting angiogenesis [21,22,28,29], and exhibiting antimicrobial properties [31]. Furthermore, as polyphenols, PACs are potent antioxidants, underscoring their significance in the treatment of oxidative stress-related skin inflammation, UV-induced skin damage, and premature aging [37,38,39].

Although the understanding of the biological activities of PACs in treating and preventing various skin disorders has advanced significantly, a comprehensive review of these results is still required. Therefore, this paper aims to summarize the main findings from in vitro and in vivo studies, focusing on the biological effects of PAC-dependent treatments for wounds, skin inflammation, acne, photodermatoses, and the prevention of UV-induced skin dysfunctions.

## 2. Results and Discussion

### 2.1. PACs—Chemical Structure and Distribution in Plants

#### 2.1.1. Chemical Structure

**Figure 1:**
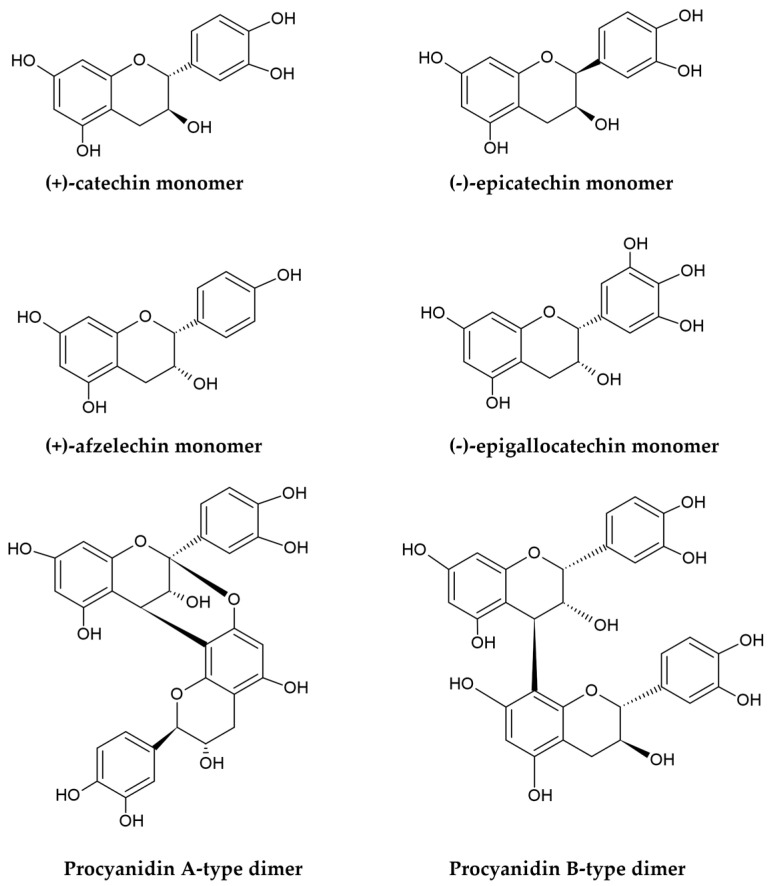
Structures of flavan-3-ols monomers and procyanidin A/B-type dimers.

PACs, also named condensed tannins, are polyphenolic plant metabolites produced through the polymerization of flavan-3-ols, primarily (+)-catechin, (−)-epicatechin, (+)-afzelechin, (−)-epiafzelechin, (+)-gallocatechin, and (−)-epigallocatechin (Figure 1) [40,41].

PACs are classified based on their molecular masses and degree of polymerization into oligomers, such as dimers, trimers, and tetramers (comprising 2–4 flavanol subunits), as well as larger polymers that can even contain more than 50 units in some cases [17,18,42]. The condensation is primarily catalyzed by polyphenol oxidases; it introduces C-C interflavan linkages (B-type bonds) within the flavan skeletons by connecting two subunits at the C4-C8 (most commonly) or C6-C8 positions. Under suitable temperature and pH conditions, additional oxidation of PACs, facilitated by radical species or polyphenol oxidases, can lead to the formation of interflavan ether bonds, designated as C-*O*-C (A-type bonds), connecting carbon atoms at the C2 and C7 or C2 and C5 positions of the monomeric scaffolds [18,43]. As a result, two types of PACs can be distinguished based on their interflavan linkages: B-type PACs, in which monomers are joined by a single B-type bond, and A-type compounds, which contain both a covalent B-type bond and an ether A-type linkage (Figure 1). Additionally, the hydroxyl groups at the C3 positions in some monomeric subunits of both A-type and B-type PACs may be esterified with gallic acid to create 3-*O*-gallates [44]. The structural diversity of PACs is further influenced by the stereochemistry of the central ring and the presence of chiral centers at the C2, C3, and C4 positions of the flavanol skeletons. Variations in interflavan linkages and spatial configurations lead to the formation of isomers [17]. For instance, the most common dimeric B-type PACs, composed of either (+)-catechin or (−)-epicatechin units, exist as eight isomers: procyanidins B_1_-B_4_ (with C4-C8 bonding) and B_5_-B_8_ (with C4-C6 linkages). Similarly, the most prevalent A-type dimers are A_1_-A_4_ (with C4-C8 and C2-*O*-C7 linkages), A_5_-A_6_ (with C4-C6 and C2-*O*-C7 bonding), and A_7_-A_8_ procyanidins (with C4-C6 and C2-*O*-C5 bonds) [18,43,44,45].

**Figure 2:**
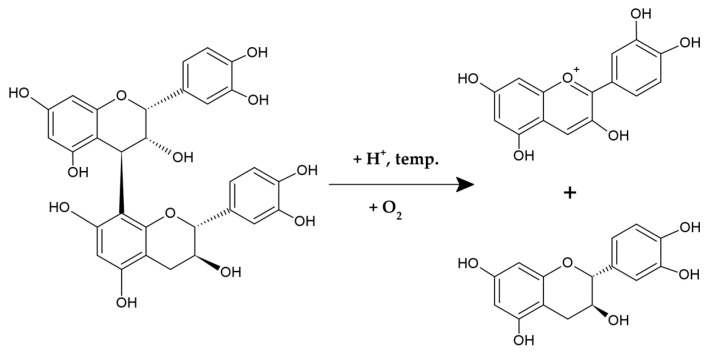
The proanthocyanidin reaction.

The term PACs refers to the depolymerization effects that occur in an acidic environment when flavan-3-ol polymers react with concentrated mineral acids, such as hydrochloric or sulfuric acids. This reaction, known as the proanthocyanidin reaction, converts the extension monomeric flavan-3-ol units into colored anthocyanins (Figure 2), which form the basis for a classical spectrophotometric assay for determining total content of PACs. Based on the structure of anthocyanins produced during this reaction, PACs can be divided into several subgroups. For example, polymers built from flavanols with a 3,4-dihydroxylation in their B-rings, such as catechin or epicatechin, yield cyanidin and are termed procyanidins. Conversely, PACs composed of specific flavonoid subunits with one or three hydroxyl groups in their B-rings generate pelargonidin or delphinidin, respectively, being referred to as propelargonidins and prodelphinidins [42,43,45,46].

The biological properties of PACs depend on their structure, primarily their polymerization degree, as well as their hydroxylation pattern, interflavan bonding, and spatial configuration, which affect their stability, bioavailability, and interactions with functional biomolecules [44,47]. Generally, due to their relatively high hydrophilicity and molecular weights, PACs have limited bioavailability after oral administration. However, they still exhibit potent biological activity within the intestine and in topical applications, thanks to their prebiotic effects and strong ability to form stable hydrogen bonds with structural and functional proteins [48,49]. The intestinal microbiota can also metabolize PACs into low-molecular-weight molecules, such as valerolactones and phenolic acids, which are more readily absorbed into the bloodstream and provide a wide range of health benefits [18,42]. The absorption rate of PACs decreases as their degree of polymerization increases; oligomers, primarily dimers, can be partially absorbed, while polymers are typically not absorbed. The systemic activities of PACs have been summarized in several reviews on their cardioprotective [17,18,45,46,47], vasodilatory [45,46], anti-inflammatory [17,18,45,46,47], antidiabetic [17,18,45,46,47], lipid-lowering [17,18,45,46,47], anti-obesity [18,45,46,47], hepatoprotective [17,18,47], neuroprotective [17,18,46,47], antimicrobial [18,45,46,47], and anticancer effects [17,18,45,46,47]. This review focuses on the topical applications of PACs and their beneficial effects in treating skin disorders.

#### 2.1.2. Distribution in Plants

PACs are the second most abundant group of natural polyphenols after lignin [17,18]. As endogenous PACs for plant protection and adaptation to both abiotic and biotic stress factors, they are accumulated in all plant organs, including stems, barks, leaves, flowers, fruits, seeds, roots, and rhizomes [45,50,51]. The distribution of PACs in plants can vary significantly due to genetic factors (interspecific variability), environmental conditions, developmental stage, and plant organs (intraspecific variability) [45,50]. Differences related to genotype have been observed in PAC profiles across various species or varieties, e.g., *Cinnamomum* [52], *Sorbus* [53], *Cotoneaster* [54], *Crataegus* [55], *Malus* [56], and *Populus* species [57]. Numerous reports have also highlighted fluctuations in PACs biosynthesis related to the vegetative stage of the plant. For instance, PACs content in fruits typically decreases during ripening [58,59,60], while it is notably intensified during leaf development [61]. There are also significant organ-specific variations in PACs levels. While fruits are often regarded as the richest source of PACs in the human diet [62], other plant organs, such as roots, rhizomes, bark, leaves, and seeds, can also accumulate similar or even higher levels [59,63,64]. For instance, grapevine seeds contain a higher PACs content compared to grapes or grapevine leaves [63,65].

Beyond quantitative variations in total content, plant sources vary in their qualitative profiles of PACs. Compounds derived from (+)-catechin and (−)-epicatechin, are most common, while PACs built of monomers with lower or higher hydroxylation, such as prodelphinidins or propelargonidins, are less frequent; however, they can even dominate in specific sources, such as *Diospyros kaki* fruits or *Senna alata* leaves, respectively [66,67]. Similarly, galloylated PACs, though rare, are abundant in specific materials, such as grape seeds [68], persimmon fruits [66], and *Vitellaria paradoxa* nuts [69]. In terms of interflavan linkages, B-type PACs are more prevalent in plants, while A-type PACs have a narrower distribution. Consequently, the abundance of A-type PACs can be found in limited species, such as *Litchi chinensis*, *Prunus spinosa*, *Diospyros kaki*, or *Laurus nobilis* [18]. The A-type/B-type PACs ratio may also vary within plant genera; e.g., A-type prevails in *Cinnamomum burmannii* and *C. verum*, while B-type dominates in *C. cassia* and *C. loureiroi* [52]. Additionally, the degree of polymerization also differentiates PACs across various species and organs. Typically, 3 to 11 units are observed, but dimers are also common, and polymers with 50 to 60 monomers have been reported [17,18,42,51,70]. The representative PACs-abundant plant species and organs are presented in Table 1.

### 2.2. Therapeutic Potential of PACs in Wound Healing

#### 2.2.1. Molecular Basis of Tissue Regeneration and Wound Healing

There are two distinct types of wounds: acute and chronic. Acute injuries typically heal within 3 months, while chronic wounds take over 12 weeks to regenerate and are at a higher risk of infections and complications, making them increasingly challenging for effective wound management [10,90].

**Figure 3:**
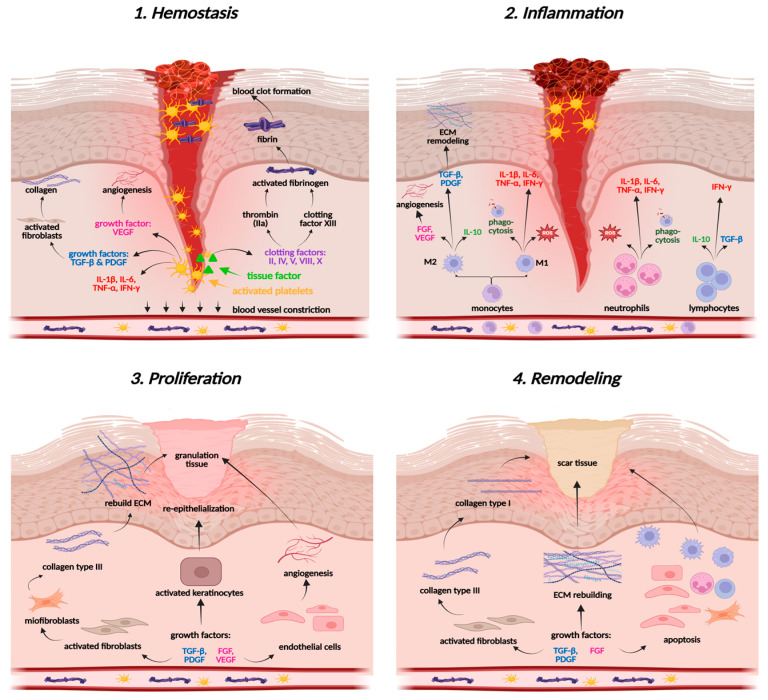
Wound healing stages. Created in BioRender. Prokop, A. (2025) https://BioRender.com/ng1u5qi.

Wound healing involves the coordinated action of various molecular mechanisms, resulting in a multi-phased cascade of events that can be categorized into four primary stages: hemostasis, inflammation, proliferation, and tissue remodeling (Figure 3). The first phase, hemostasis, prevents excessive blood loss by platelet activation and aggregation involving collagen, a crucial element of the ECM [90,91]. Platelets are responsible for recruiting immune cells and releasing granular contents, such as platelet-derived growth factor (PDGF) and transforming growth factor beta (TGF-β). Collagen, platelets, thrombin, and fibrinogen form a fibrin clot, initiate inflammatory processes in the wound area, and recruit further factors and cytokines [10,90].

The next stage of the wound healing process is inflammation, which aims to cleanse the wound area of pathogens and necrotic debris, thereby stimulating mechanisms crucial for tissue repair. A dysfunctional inflammatory response can impede tissue regeneration and lead to the development of a chronic condition [10]. In the damaged area, pro-inflammatory cytokines (IL-1β, IL-6, TNF-α), pro-inflammatory and pro-oxidative enzymes like nitric oxide synthase (iNOS), cyclooxygenase-2 (COX-2), as well as interferon gamma (IFN-γ), activate the first cellular responders—neutrophils, which start oxidative burst, release reactive oxygen species (ROS) and cytokines, and recruit monocytes to enhance the immune response and support phagocytosis (Figure 3) [10,90]. Monocytes, when triggered by TNF-α and IFN-γ, differentiate into pro-inflammatory macrophages (M1), while those stimulated by IL-4, IL-10, IL-13, or TGF-β differentiate into anti-inflammatory macrophages (M2) [91]. The latter ones are crucial for tissue formation. They secrete anti-inflammatory cytokines and promote growth factors, including VEGF (vascular endothelial growth factor) and FGF (fibroblast growth factor), which focus on angiogenesis and tissue formation, as well as TGF-β and PDGF, which regulate the functions of fibroblasts and collagen [92,93]. M2 cells also secrete antioxidant enzymes, such as superoxide dismutase (SOD), catalase (CAT), and glutathione peroxidase (GSH-Px), re-establishing redox balance in wound tissues [94]. The cooperation of macrophages, cytokines, and growth factors provides a smooth transition from the inflammatory phase to the proliferation in acute wounds, while it is markedly disturbed in chronic wounds, leading to persistent inflammation [92].

The proliferative phase involves angiogenesis, fibroplasia, and reepithelialization, vital for wound contraction [92]. During angiogenesis, the functions of endothelial cells in the growth, proliferation, and migration of skin cells are orchestrated by growth factors, such as VEGF, FGF, PDGF, and TGF-β. The expansion and movement of keratinocytes, fibroblasts, and endothelial cells aim to replace the clot, rebuild the skin barrier, and form granulation tissue. Fibroblasts produce an immature collagen network composed of type III fibers, forming the ECM—the basis for further wound healing [10,90]. Reepithelialization (epidermal regeneration) is driven by keratinocytes that form a new epidermal layer. This phase restores tissue architecture and facilitates final wound remodeling [92,95].

During the remodeling stage, the ECM is modified and reorganized to enhance tissue strength, with collagen III being replaced by collagen I under the influence of TGF-β and VEGF. Furthermore, anti-inflammatory cytokines (IL-10, TGF-β) control tube formation and prevent excessive angiogenesis [92,95].

#### 2.2.2. PACs—Wound Healing Activity

The accumulated in vitro, in vivo, and ex vivo research suggests that PACs may promote wound healing by targeting all stages of the process, including hemostasis, inflammation, proliferation, and remodeling. Their influence in the early phases is mainly manifested through the promotion of keratinocyte and fibroblast proliferation and migration. For instance, such an effect has been shown for PACs-enriched extracts from grape (*Vitis vinifera* L.) seeds and *Stryphnodendron adstringens* bark, as well as for isolated compounds, such as dimeric procyanidin B2 (PB2) and trimeric cinnamtannin B1 [19,20,26,28,29,30,32]. PACs also enhance neutrophil infiltration into the wound environment and accelerate wound cleansing, thereby initiating the inflammatory stage. Additionally, PACs reduce the secretion of pro-inflammatory enzymes, such as iNOS and COX-2, and target the activity of TNF-α, thereby regulating the intensity of inflammatory responses and preventing excessive and chronic inflammation [21]. Furthermore, PACs, as polyphenols, exhibit potent antioxidant properties and effectively maintain the oxidative balance within the wound area by scavenging ROS, downregulating intracellular ROS levels, and enhancing antioxidant protection through glutathione regulation [21,24,25,31]. In addition, PACs, such as dimeric PB2, effectively activate the Nrf2 signaling pathway and, as a consequence, stimulate the activity of antioxidant enzymes, including SOD and CAT, supporting a transition from the inflammatory to the proliferative phase [22]. PACs also exhibit pro-angiogenic properties by stimulating VEGF and promoting endothelial cell migration, thereby contributing to neovascularization. Numerous studies indicate that PACs, especially PB2, cinnamtannin B1, PACs-enriched *Mallotus philippinensis* bark extract, and grape seed extract, can improve not only the quantity of new tubes but also the qualitative characteristics of capillaries [22,24,28,29]. Studies have also demonstrated a significant impact of oligomeric PACs on later stages of wound healing, particularly on ECM remodeling and collagen control. The results suggest that PACs may have strong anti-fibrotic potential and protective effects against excessive hypertrophy and improper scar formation [27,32]. Notably, significant contributions and innovations in terms of activity were presented in studies of new formulas for topical administration of PACs, such as nanoparticles and hydrogels, which additionally increased hemostatic activity, improved tissue integrity, and exhibited antibacterial activity of crude compounds and extracts [30,31,96].

Despite significant preclinical data, many in vitro studies have not advanced to animal models, and only one human trial has been performed. A randomized, double-blind study involving 40 patients compared a cream with 2% grape seed extract with placebo, showing accelerated wound healing. However, the lack of phytochemical data on the extract (undisclosed content and structure of PACs) hinders definitive conclusions about their clinical efficacy, thereby identifying the most critical issue for future validation [96]. The second key knowledge gap remains in understanding how PACs affect the wound healing factors, such as TGF-β, IFN-γ, and FGF, as well as IL-10 and the differentiation of M1/M2 macrophages, which are crucial for resolving inflammation and preventing excessive angiogenesis. The molecular basis of the PAC action on collagen secretion and maturation should also be explored in more detail. Eventually, the structure-activity relationships of PACs in wound healing require experimental verification.

The subsequent parts of this section discuss the potential of PACs in wound healing, highlighting their biological mechanisms, efficacy, and innovative formulations, particularly for topical application. Detailed data from in vitro and in vivo studies are provided in Supplementary Materials Table S1.

The in vitro studies of Tsuruya et al. [19] and Kisseih et al. [20] suggest that PACs and PAC-enriched extracts may play a crucial role in stimulating wound healing by targeting the proliferation stage (supporting keratinocyte differentiation and fibroblast proliferation) and restoring redox balance in the wound area. PACs from commercially available product Leucoselect^®^ of grape seed (*Vitis vinifera* L.) standardized extract, containing not less than 95.0% of PACs, accelerated the 3T3-L1 mouse fibroblasts proliferation in a dose-dependent manner, sustaining their activity even under UV-irradiation; additionally, PACs exerted antioxidant effects by reducing H_2_O_2_-induced intracellular oxidative stress [19]. Furthermore, the PACs-containing *Combretum mucronatum* leaf aqueous extract stimulated the differentiation of pNHEK keratinocytes, while inhibiting the proliferation of HaCaT keratinocytes, thus demonstrating notable selectivity towards various cell lines [20]. Similarly, among several PACs in the extracts, only PB2 affected cellular differentiation, while other compounds (procyanidins B5, C1, and D1) had no effect [20].

The study by Chen et al. [21] has been revealed that the commercially available PAC-rich grape seed extract (GSP) can downregulate excessive ROS production in human umbilical vein endothelial cells (HUVECs) in vitro, thereby preventing the adverse effects of oxidative stress on wound closure. GSP protected cells from the prooxidant action of H_2_O_2_ by significantly lowering ROS level, and increasing activity of antioxidant enzymes, particularly manganese SOD and CAT, by activating the p-JNK/FOXO3a signaling pathway. Furthermore, GSP mitigated the excessive mitophagy, which is vital for wound healing, by downregulating the expression of mitophagy proteins PINK1, Parkin, and LC3-II, while upregulating the expression of antioxidant protein p62 [21].

The significant role of the antioxidant activity of PACs in diabetic wound healing, particularly during angiogenesis, is highlighted by the in vitro study of Fan et al. [22] on endothelial progenitor cells (EPCs) in the high glucose (HG) model. In the study, PB2 significantly increased *CAT* and *NQO-1* gene expression, thereby regulating the Nrf2 signaling pathway in a dose-dependent manner, where Nrf2 is a transcription factor essential for cellular defense against oxidative stress. As a result, PB2 counteracted the HG-induced pro-oxidative effect, preserving proper angiogenic functions of EPCs, reducing apoptosis, promoting migration, and accelerating wound closure in vitro [22]. The wound-healing potential of PB2 has been further confirmed in vivo in a streptozotocin (STZ)-induced diabetic C57/BL6 mice, with a significant reduction in ROS level and an increase in circulating EPCs observed, which translated into promoted wound healing and angiogenesis [22].

The impact of PACs on angiogenesis and VEGF transcription and expression has also been investigated in HaCaT keratinocytes under H_2_O_2_-induced stress and TNF-α-induced inflammation [23,24]. ActiVin, the novel commercial grape seed extract (GSPE), containing ca. 74% of PACs, upregulated the protein and mRNA expression of VEGF in a dose-dependent manner, both under the H_2_O_2_-induced stress and independently. Additionally, GSPE enhanced the TNF-α-induced release of VEGF protein [23]. The topical application of GSPE in vivo (BalbC mouse dermal wound model) accelerated wound contraction and closure, as well as mitigated the oxidative stress by increasing the glutathione disulfide (GSSG)/glutathione (GSH) ratio. Additionally, GSPE significantly stimulated the expression of VEGF and tenascin, an ECM glycoprotein, thereby improving the histological structure of the wound and vascularization [24].

The complex wound-healing potential of PACs has been studied for extracts of various Alaskan berries, including bog blueberry (*Vaccinium uliginosum* L.), crowberry (*Empetrum nigrum*), and lingonberry (*Vaccinium vitis-idaea* L.), containing approximately 22–55% dw of PACs [25]. Each extract enhanced the migration of human dermal fibroblasts (HDFa) into the wound area in vitro. PACs reduced ROS levels similarly to the positive control, dexamethasone (DEX), and decreased NO production in LPS-stimulated mouse macrophages (RAW264.7), with the most potent effect observed for the lingonberry extract; however, this effect was less potent than that observed for the positive control. Additionally, PACs demonstrated their anti-inflammatory potential by markedly down-regulating the gene expression of key pro-inflammatory factors, such as COX-2 (the effect of the bog blueberry fraction was comparable to that of DEX) and iNOS (the effect of the lingonberry and crowberry fractions was marginally less potent than that of DEX) [25].

Enhanced fibroblast migration and accelerated wound closure have also been observed for PACs-rich fractions from lingonberry leaves (99 mg/g dw of PACs, with procyanidin A1 as principal compound) in the model of human foreskin fibroblasts (HFF) in vitro [26]. Interestingly, migration was potentiated only at relatively low levels of PACs, with a reverse effect at higher concentrations, which may indicate a potential for counteracting hypertrophy. The transdermal PAC-delivery systems have also been studied, with those composed of three polymers (methylcellulose, hydroxyethyl cellulose, and polyethylene glycol 400) releasing up to 56.5% of the extract in 4 h with zero-order kinetics [26].

Potential of PACs to regulate the remodeling phase of wound healing in vitro has been demonstrated for oligomeric procyanidins (OPCs) from grape seeds in human dermal fibroblasts (Hs27) [27]. OPCs diminished TGF-β1-induced procollagen I secretion and decreased its incorporation into the ECM in a dose-dependent manner, thereby counteracting the profibrotic effect [27]. OPCs also modulated the transportation and secretion of procollagen, suggesting their ability to prevent the formation of hypertrophic scars. On the other hand, in combination with ascorbic acid, which is a supporting factor for collagen maturation, OPCs significantly reduced both procollagen secretion and intracellular accumulation. Ultimately, OPCs reduced the availability of collagen in the ECM, without affecting collagen synthesis or disrupting the natural tissue regenerative processes, potentially by regulating degradation or inhibiting translation [27].

Studies on the PACs-rich *Mallotus philippinensis* bark extract (EMPB), which contained cinnamtannin B1, a trimeric compound, have revealed markedly promoted proliferation and migration of NIH-3T3 fibroblasts, and migration of NHEK keratinocytes and HAECs in vitro, indicating involvement of EMPB in ECM reconstruction, wound closure, and angiogenesis [28]. Moreover, EMPB suppressed the proliferation of RAW264.7 macrophages, thereby limiting the excessive inflammatory response. EMPB also stimulated the proliferation and migration of KUM6 mesenchymal cells. Cinnamtannin B1, the primary constituent of EMPB, exhibited a similar effect, also promoting either proliferation or migration of KUM6 cells [28]. Topical treatment with EMPB [28] and cinnamtannin B1 [29] of diabetic C57BLKS/Jlar- + Lepr^db^/+ Lepr^db^ mice has resulted in significant and dose-dependent pro-regenerative and proangiogenic effects, accelerated wound closure, and stimulated formation of new capillaries and granulation tissue, thereby providing an optimal dermal architecture for the remodeling phase of wound healing [28,29].

Apart from investigating the effects of crude substances, some research groups have also examined the impact of pharmaceutical formulations on the wound-healing potential of PACs. For instance, Orłowski et al. [30] compared the activity of bimetallic gold-silver nanoparticles modified with procyanidin B2 (ProNPS) to that of PB2 alone in a combined in vitro and in vivo study. In general, the effects of the formulated compound were more pronounced. ProNPS significantly stimulated the migration of HaCaT keratinocytes and upregulated the expression of critical genes for wound healing and tissue regeneration. Thereby, ProNPS promoted keratinocyte proliferation, reinforced the skin barrier function, and facilitated the ECM remodeling. Moreover, PB2-loaded nanoparticles significantly reduced the area of excisional wounds in C57BL6 mice treated topically by triggering neutrophil chemotaxis and upregulating the PDGF-β expression. This indicated the stimulatory effect of PB2 on the smooth transition of wound healing from the inflammatory phase to the proliferative stage [30].

For analyzing the effectiveness of novel PACs-rich formulations, the oligomeric PACs from grape seeds (OPCG) have been incorporated into the PBO/PBOF hydrogel (polyvinyl alcohol, borax, OPCGs, with or without ferric ion), and tested in the murine fibroblast cell line L929 [31]. The OPCG-loaded hydrogels exhibited potent antioxidant activity by effectively scavenging ROS and protecting fibroblasts from oxidative stress. Furthermore, potent antibacterial activity against *Escherichia coli* and *Staphylococcus aureus* was shown, with a synergistic effect with near-infrared (NIR) light, suggesting that the OPCG-loaded hydrogel may be beneficial for preventing bacterial infection in chronic wounds. The hydrogels also displayed significant hemostatic potential in vitro, indicating their ability to improve clotting in the wound area without risk of excessive thrombosis. In vivo tests with animal models confirmed the high efficacy of the tested formulations in reducing bleeding and hemostatic time. The treatment of wounded Kunming mice with OPCG-loaded hydrogels also resulted in a significantly increased rate of wound closure, especially when combined with NIR light, accelerated epidermal regeneration, significantly enhanced collagen deposition, and improved the structure of collagen fibers [31].

Topical application of a gel loaded with 1% extract of *Stryphnodendron adstringens* bark, containing monomeric and oligomeric (epi)gallocatechins and their methylated and galloylated derivatives (no exact content given) has been tested on skin wounds in streptozocin-induced diabetic Wistar rats [32]. This treatment resulted in increased keratinocyte proliferation and migration, along with a significant rise in VEGF and COX-2 expression during the initial phase of wound healing, thereby promoting angiogenesis and re-epithelialization. In contrast, the expression of these factors declined after this time, enabling a smooth transition to the subsequent phases. The gel also had a beneficial effect on collagen maturation and, consequently, ECM remodeling, as it stimulated the replacement of type III collagen fibers by the thicker type I collagen (mature collagen) [32].

### 2.3. Therapeutic Potential of PACs in Inflammatory Skin Diseases

#### 2.3.1. Molecular Basis of Skin Inflammation

The pathogenesis of inflammatory skin disorders is complex and involves interactions among skin cells, immune cells, and signaling factors [97]. Skin inflammation can arise from exposure to external stimuli, such as UV radiation, allergens, or mechanical injury, along with autoimmune responses and genetic predispositions. Inflammatory skin diseases activate both innate and adaptive immune systems, which can negatively impact the skin tissues. The progression of inflammation-related skin problems involves pro-inflammatory mediators, such as cytokines, which affect the keratinocytes and fibroblasts, amplifying pro-inflammatory gene expression and altering the autoimmune response. The differentiation of autoimmune skin disorders is primarily determined by specific cytokine and immune cell profiles participating in the inflammatory response and its intensity. Inflammatory conditions of the skin can also be classified as acute or chronic based on their duration [98]. Acute inflammation, a short-term physiological response to a stressor, resolves once the cause is removed. Chronic inflammation is a more complex condition that can originate from acute inflammation. The most widespread skin diseases, which arise from a dysregulated inflammatory response driven by the overactivation of immune pathways and a dysfunctional skin barrier, are atopic dermatitis and psoriasis [97,98,99]. Additionally, exposure to UV radiation initiates UV-induced skin inflammation. It penetrates the skin, inducing DNA alterations and cellular damage, particularly in keratinocytes, resulting in an exaggerated inflammatory response—a key early event in the onset of photoaging, chronic actinic dermatitis, or photocarcinogenesis [100].

##### Psoriasis

**Figure 4:**
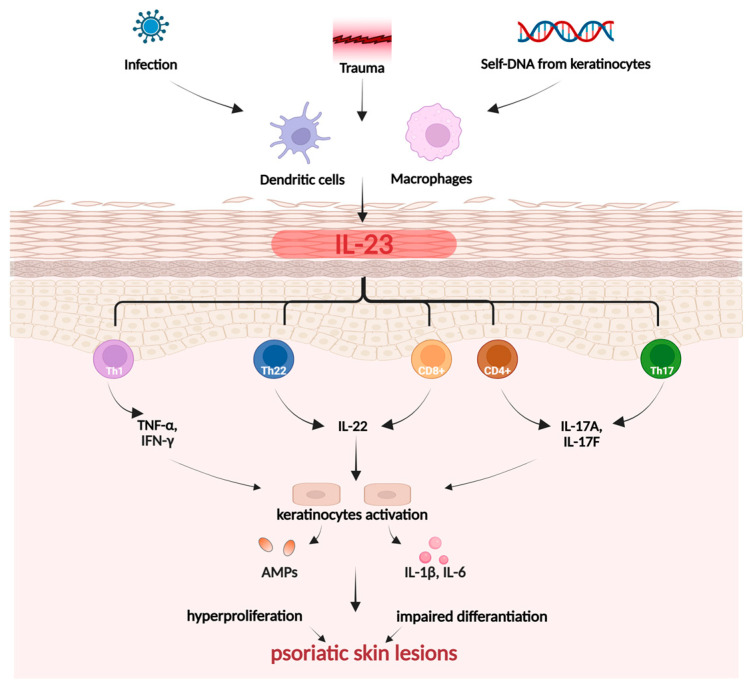
Pathogenesis of psoriasis. Created in BioRender. Prokop, A. (2025) https://BioRender.com/zswpm4d.

Psoriasis is a chronic, autoimmune, and inflammatory skin disease affecting approximately 2–3% of individuals globally [98]. It is triggered by a combination of external, genetic, and immunological factors, with a high degree of complexity and pathogenesis closely linked to T-helper cells 17 (Th17) (Figure 4).

During the initiation phase of psoriasis, trauma can induce inflammation of the skin, an event known as the Koebner phenomenon [101]. In response to the disrupted function of the skin barrier and overactivation of the immune system, keratinocytes begin to produce chemokines. This action attracts immune cells, primarily monocytes/macrophages, as well as Th cells, leading to boosted expression of pro-inflammatory cytokines, mainly IL-17, IL-23, and TNF-α. Simultaneously, the innate immune system initiates a response involving Th17 and Th22 [101,102]. The state of chronic inflammation leads to excessive keratinocyte proliferation and impaired differentiation, resulting in the formation of skin lesions—the hallmark of psoriasis [101]. The development of the most prevalent psoriasis subtype, known as plaque psoriasis, is closely linked to the TNF-α-IL23-Th17 axis. The Th17, Th22, and T cells (CD4+ and CD8+) are responsible for producing crucial cytokines, including IL-17A, IL-17F, IL-22. Keratinocytes, stimulated by IL-17, undergo hyperproliferation and release pro-inflammatory cytokines IL-1β and IL-6. Meanwhile, IL-22 contributes to skin barrier impairment and blocks keratinocyte differentiation. As a result, keratinocytes exhibit malfunction, leading to increased stratum corneum (hyperkeratosis), impaired cornification, and epidermal thickening. Modulation of the positive feedback loop in chronic inflammation also depends on the regulation of Th1 and Th2 cytokines by Janus kinase (JAK)-STAT signaling pathways [101,102].

##### Atopic Dermatitis

**Figure 5:**
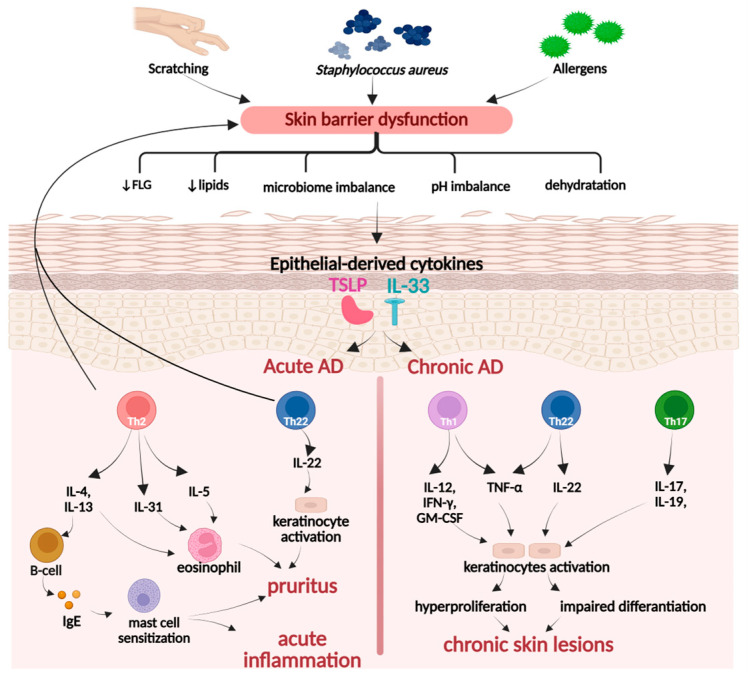
Pathogenesis of atopic dermatitis. Created in BioRender. Prokop, A. (2025) https://BioRender.com/kam0kqu.

Atopic dermatitis is a skin disorder with a complex pathophysiology, primarily initiated by a combination of genetic factors that lead to a defective skin barrier and external triggers, such as allergens and *Staphylococcus aureus* colonization on the skin (Figure 5). It is distinguished into acute or chronic conditions, accompanied by multiple symptoms, such as erythema, edema, papules, vesicles, intense pruritus, and lichenification, which arises as a consequence of persistent pruritus and scratching [103].

Moreover, in the case of acute dermatitis, overactivated Th2 and Th22 cells downregulate the expression of critical skin proteins, primarily filaggrin (FLG), resulting in increased skin permeability to pathogenic and allergic factors. An impaired epidermal barrier triggers keratinocytes to release thymic stromal lymphopoietin (TSLP), which simultaneously promotes the secretion of Th2 and Th22 cells. In the chronic disease type, the predominant immune pathways involve Th1 rather than Th2, as well as the overactivity of the Th17/Th22 axis. The coordinated action of cytokines, such as IFN-γ, IL-17, IL-22, and TNF-α, contributes to hyperproliferation and decreased differentiation of keratino-cytes, resulting in epidermal thickening, itching, and development of chronic skin lesions [103,104,105].

##### UV-Induced Skin Inflammation

**Figure 6:**
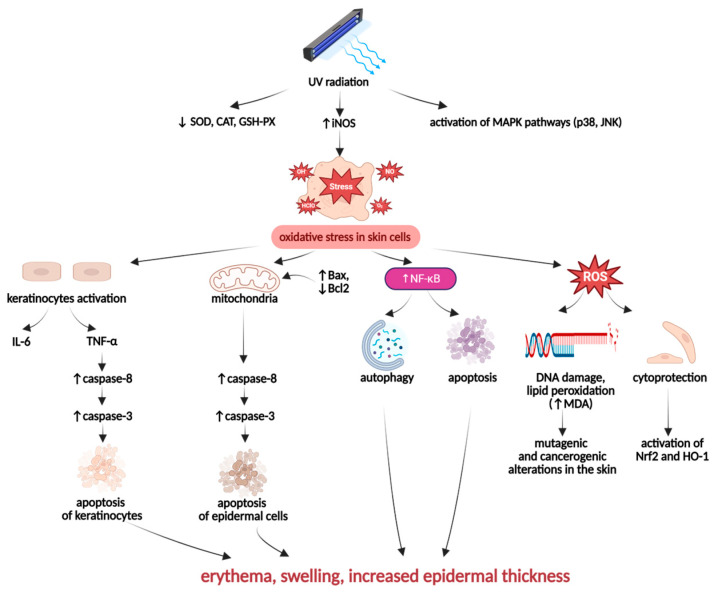
Pathogenesis of UV-induced skin inflammation. Created in BioRender. Prokop, A. (2025) https://BioRender.com/mgmof3w.

Excessive and prolonged exposure of the skin to ultraviolet (UV) radiation is a significant risk factor for the development and progression of various skin disorders. UVA and UVB can affect keratinocytes, inducing DNA damage and oxidative stress-induced inflammation (Figure 6). These processes contribute to skin impairment and constitute one of the crucial risk factors in photoaging, skin carcinogenesis, the development of wrinkles, and epidermal pigmentation [106,107].

UV-irradiated skin cells may be affected by an oxidative imbalance resulting from the suppressed activity of antioxidant enzymes, such as SOD, GSH-Px, and CAT, and the overactivity of pro-oxidative enzymes like iNOS. Moreover, UV-irradiation can also modify the expression of mitogen-activated protein kinases (MAPKs), including p38 kinase and c-Jun N-terminal kinase (JNK), which contribute to the induction of oxidative stress in skin cells [97,107]. The UV-irradiation-induced intracellular redox imbalance leads to the overgeneration of ROS, which can cause toxic, mutagenic, and carcinogenic alterations in skin cells [105]. By activating nuclear factor-κB (NF-κB), UV radiation can stimulate cell death processes, especially autophagy and apoptosis in skin tissues. Furthermore, UV-irradiated keratinocytes are stimulated to produce pro-inflammatory cytokines, especially IL-6 and TNF-α, which, acting together with ROS, contribute to an excessive tissue inflammation. The primary hallmarks of UV radiation-promoted skin inflammation are erythema, swelling, and increased epidermal thickness [108,109].

#### 2.3.2. PACs—Anti-Inflammatory Activity

Although studies addressing the effects of PACs in skin disorders related to dysregulated inflammatory response are relatively scarce, they point to the promising potential of these compounds [33,34,35,36]. Findings from in vitro and in vivo experiments confirm that PACs can modulate the inflammatory response by targeting numerous pro-inflammatory pathways, including those involving Th17 lymphocytes, crucial for the pathogenesis of psoriasis, and Th2 lymphocytes, which are involved in atopic dermatitis. For instance, dimeric PACs have been demonstrated to significantly reduce the activity of immune cells (eosinophils), as well as the serum levels of Th2 cytokines (IL-4, IL-5, IL-3) and immunoglobulin E (IgE) after topical application in NC/Nga mice with induced atopic dermatitis-like skin lesions. The effects of PACs were comparable to those of hydrocortisone, which underscores their potential as anti-inflammatory agents for treating atopic dermatitis [36]. Significant inhibition of excessive inflammatory responses on the cellular and molecular levels has also been proven for oligomeric PACs in animal models, including an imiquimod-induced psoriasis-like mouse skin model. PACs effectively reduced the levels of Th17 cytokines (IL-17, IL-23), regulated the expression of pro-oxidant and pro-inflammatory genes and proteins, and diminished neutrophil infiltration, indicating multidirectional anti-inflammatory activity [33,34,35]. Furthermore, in all in vivo studies of psoriatic and atopic skin models, significant improvement in skin condition and alleviation of clinical symptoms were noted, including effective inhibition of keratinocyte hyperproliferation [33,34,35,36]. Notably, researchers have recorded the dependence of PACs effectiveness in managing inflammatory skin conditions on their structure, with dimers and trimers demonstrating the most advantageous efficacy [35,36]. Despite the reported data being promising, the limited number of studies poses a challenge to translating PACs into routine phytotherapy for psoriasis and atopic dermatitis. The critical knowledge gap is the lack of human trials; however, preclinical data also require completion through a deeper insight into molecular mechanisms and a wider range of tested extracts and pure compounds. The latter is critical for ultimately verifying the structure-activity relationships of PACs in treating skin inflammation.

The details of the investigations performed to date on this topic are described below and summarized in Supplementary Materials Table S2 [39].

Results of the in vitro study by Zhao et al. [33] in the TNF-α-induced psoriasis-like HaCaT keratinocyte model revealed that PACs may target two key psoriasis mechanisms related to keratinocyte function: pro-inflammatory response and hyperproliferation. It has been shown that PACs (no specific data provided regarding origin and content) significantly inhibited the PI3K/AKT signaling pathway, which is responsible for stimulating the production of pro-inflammatory cytokines (IL-17 and IL-23), typical of Th17 cells, mitigating cytokine-driven inflammation and averting keratinocytes from hyperactivation. PACs also acted anti-inflammatory by regulating the antioxidant defense system through targeting HO-1 (heme-oxygenase-1) expression, concurrently increasing levels of endogenous antioxidants (SOD, CAT, GSH). A multi-directional action of PACs disrupted the pathological inflammatory network and excessive skin cell growth, typical of psoriasis, by targeting all three pathways: PI3K/AKT, HO-1, and JAK/STAT [33].

Toda et al. [34] have demonstrated that an oligomeric PAC (B-type catechin octamer, RRP) from red-kerneled rice hulls acts as a highly efficient, non-competitive and non-chelating inhibitor of rat and human 5-LOX, compared to catechin and zileuton (a potent synthetic 5-LOX inhibitor). Moreover, RRP has been shown to target the early stages of inflammation in vivo by effectively inhibiting microsomal prostaglandin E synthase-1 (PGES-1), thereby regulating prostaglandin E2 (PGE2) synthesis, while having little to no inhibitory effect on other pro-inflammatory enzymes, such as COX-1, COX-2, and mPGES-2. Indeed, topical application of RRP exerted an anti-inflammatory effect in an imiquimod (IMQ)-induced psoriasis mouse, as evidenced by decreased levels of leukotriene B4 (LTB4) and modulation of the expression of psoriasis-related genes, including 12-HETE, *IL-17*, *IL-22*, *S100a9*, and *Krt1b*. This action leads to improved disease symptoms—reduced epidermal hyperplasia and skin thickness [34].

The anti-inflammatory potential has also been evidenced for oligomeric PACs from peanut skin in LPS-stimulated human cultured monocytes THP-1 [35]. A significant reduction was observed in the levels of IL-6 and TNF-α; furthermore, nine A-type and one B-type PACs differing in degree of polymerization have been compared, with the conclusion that dimers and trimers exhibit superior effects over monomers and tetramers [35].

Evidence provided by Park et al. [36] has shown that PACs from the roots of *Rosa multiflora* Thunb., including procyanidin B3 (PB3) and ent-guibourtinidol-(4β → 6)-catechin (RM1), act as immunomodulators in vivo, suppressing Th2 lymphocyte response, alleviating allergic reactions and inflammatory processes, which can be beneficial for treating atopic dermatitis. After 4 weeks of topical treatment with a cream containing 1% PACs, the total skin severity score (sum of individual scores for dryness, erythema, excoriation, edema, erosion, hemorrhage, and scaling) was significantly reduced in the NC/Nga mice, with the effects comparable to those of 1% hydrocortisone cream (a corticosteroid commonly used in treating dermatitis). Furthermore, PB3 and RM1 decreased levels of IgE and eosinophils in serum (RM1 was especially potent). PB3 and RM-1 also prevented the overproduction of NO and COX-2, targeting the expression of iNOS and COX-2 at both the transcriptional level and during protein translation and/or degradation, thereby disrupting the release of pro-inflammatory cytokines and counteracting inflammatory and oxidative damage in the skin [36].

#### 2.3.3. PACs—Photoprotective Activity

The investigations on the photoprotective potential of PACs performed to date, although scarce [37,38,39], emphasize that the antioxidant activity of PACs is crucial for their ability to prevent UV-induced skin damage, especially the inflammation-related effects of excessive irradiation. The multi-vector action of PACs, manifested in restoring oxidative balance in skin cells, is not only related to the reduction in intracellular ROS levels or enhancement of endogenous antioxidant defense, leading to improved resistance and increased survival of skin cells, but also to the action at the level of MAPK associated with UV-induced inflammatory response [37,38,39]. Despite extensive analysis, the precise mechanisms remain incompletely explored. There is a significant knowledge gap regarding how antioxidant enzymes are regulated, whether directly, indirectly through gene expression, or via other pathways. Moreover, limited in vivo data from animal models exist to confirm in vitro results and advance toward clinical application.

The subsequent parts of this section and the Supplementary Materials Table S3 provide a summary of the findings from in vitro and in vivo experiments conducted to evaluate the photoprotective activity of PACs.

Shi et al. [37] have documented the protective effects of persimmon (*Diospyros kaki* Thumb.) oligomeric PACs (DOP) against UV-induced damage in HaCaT keratinocytes and BALB/c mice. DOP treatment restored the endogenous antioxidant defense system in vitro and in vivo by reducing levels of ROS and increasing concentrations of SOD and GSH by upregulating the Nrf-2/HO-1 axis. Topical treatment of mice with DOP significantly reduced UV-B-induced epidermal thickness and markedly relieved swelling. Additionally, DOP inhibited the phosphorylation of the p65 subunit, thereby suppressing the activation of the NF-κB and MAPK pathways and reducing the production of IL-6 and TNF-α. DOP also reversed the cytotoxic effect of UV-B and protected skin cells from apoptosis, downregulating the expression of pro-apoptotic and anti-apoptotic proteins both in vitro and in vivo [37].

Chen et al. [38] have provided evidence that PACs of lotus (*Nelumbo nucifera* Gaertn.) seedpod (a mixture of B-type monomers and dimers), encapsulated in nanoliposomes, protect HFF in vitro against UV-B-induced oxidative stress by upregulating the secretion of SOD. Moreover, the inhibition of tyrosinase by PACs, with a more potent effect than the powerful antioxidant vitamin C, along with their increasing effects on skin cell viability, suggested their ability to inhibit melanogenesis and limit UVB-induced secondary skin damage [38].

Matito et al. [39] have shown that PACs-containing fractions from grape pomace downregulated both basal and UVB- or UVA-induced intracellular ROS generation in HaCaT cells, with the effects dependent on the dose, degree of polymerization and galloylation. The effectiveness of PAC fractions increased with the size of PACs and their esterification, from inactive nongalloylated monomers to the most efficient oligomers, with a mean polymerization degree of 2.7–3.7 and an esterification level of 25–31%. The most active PAC fractions also significantly reduced activation of MAPKs (p38 and JNK1/2). Interestingly, in the case of JNK1/2 activation, some synergy for the combined fraction containing both monomeric and oligomeric PACs, as well as their galloylated derivatives, was observed. The observed ability of PACs to target UV-induced MAPK activation and ROS generation in vitro suggests their promising potential as protective agents against UV- and oxidative-induced skin cell apoptosis and photoaging, warranting further verification in vivo [39].

## 3. Materials and Methods

The literature review was performed by searching PubMed, Scopus, and Web of Science databases for original articles published between January 2000 and August 2025. For complementary research, Google Scholar has been used. The following keywords and combinations were checked: “proanthocyanidins”, “proanthocyanidins and skin”, “proanthocyanidins and keratinocytes”, “proanthocyanidins and fibroblasts”, “proanthocyanidins and wound healing”, “proanthocyanidins and atopic dermatitis”, “proanthocyanidins and psoriasis”, “proanthocyanidins and photoprotection”, “proanthocyanidins and skin and UV”, “proanthocyanidins and UV and inflammation”, “proanthocyanidins and inflammation and skin”, “wound healing”, “psoriasis”, “atopic dermatitis”, and “UV and skin and inflammation”. The inclusion criteria were: (a) original articles published in English in peer-reviewed scientific journals; (b) articles presenting data from in vitro or in vivo studies evaluating the pharmacological effects of proanthocyanidins and proanthocyanidin-rich plant products, especially after topical application, in the context of inflammation-related skin conditions, such as wound healing or atopic dermatitis or psoriasis or UV-induced skin inflammation, including photoprotective activity; (c) articles reporting meaningful information on the chemical structure and quantity of proanthocyanidins in the tested plant products; (d) providing data on the distribution and concentration of proanthocyanidins in various plant materials; (e) providing evidence for significant contribution of proanthocyanidins to the observed biological effects; (f) summarizing information on the molecular basis of wound healing and inflammation-related skin diseases, including psoriasis, atopic dermatitis, and UV-induced skin inflammation. Specific exclusion criteria were implemented to eliminate studies being out of the scope of the present review or lacked relevant data, such as: (a) articles presenting data from studies conducted in models not relevant to skin tissues; (b) providing data from in vivo studies with a non-epidermal route of administration; (c) research focused on diseases of non-inflammatory etiologies (skin cancer or photo-aging); (d) papers that lacks experimental data; (e) papers in languages other than English. In total, 109 scientific articles were selected using these inclusion and exclusion criteria, following a comprehensive manual examination of each paper. This examination was conducted firstly by reviewing the paper’s keywords, title, and abstract, and then reading the entire article.

## 4. Conclusions

This review consolidates the recent 25 years of research on the biomolecular mechanisms, pharmacological effects, and phytochemical aspects of PACs in the context of treating wounds, psoriasis, atopic dermatitis, and UV-induced skin inflammation.

Considering the available data, the foundation for applying PACs as topical agents in modern phytotherapy is most strongly supported by their wound-healing effects. Research conducted on various skin cell lines and animal models has demonstrated that PACs affect all phases of wound healing and significantly accelerate wound closure. Their antioxidant properties play a crucial role in the wound repair process; PACs activate signaling pathways (such as Nrf), enhance the activity of antioxidant enzymes (like SOD and CAT), downregulate ROS levels, modulate the inflammatory phase of wound healing, and promote its transition to the proliferative stage. Additionally, PACs facilitate wound healing by promoting hemostasis, clearing debris and pathogens from the wound area, stimulating angiogenesis, and supporting epidermal reconstruction through increased migration and proliferation of dermal cells. They also contribute to the formation of granulation tissue, ECM remodeling, and collagen maturation. However, despite the promising preclinical data, the lack of clinical trials for properly defined PACs and PAC-rich plant extracts necessitates further investigation to provide precise therapeutic targets for their use in humans.

Clinical trials are also necessary to verify the potential of PACs as topical agents for treating immune-mediated skin inflammation induced by UV irradiation. In this case, however, additional preclinical data are needed due to the relatively limited number of studies on this topic to date. Nonetheless, PACs have demonstrated the ability to modulate key molecular signaling pathways and cellular functions associated with psoriasis and atopic dermatitis, such as those involving Th17 and Th2 lymphocytes. By affecting these pathways, PACs can impact keratinocyte proliferation and migration, counteract excessive keratosis and abnormal skin structure, and help alleviate symptoms of these diseases, such as redness, scaling, and dryness.

The primary knowledge gap in the use of PACs for treating skin conditions lies not only in the lack of clinical data but also in the understanding of their structure-activity relationships. Some reports suggest that dimers and trimers exhibit superior activity compared to monomers or polymers, and that galloylation may have beneficial effects. However, systematic studies exploring a broader range of pure compounds are necessary to identify the most effective PACs and determine the optimal profiles of plant extracts for use as topical agents in skin disorders.

Furthermore, there is a significant need to enhance research on delivery systems of PACs to improve their effectiveness and feasibility. Current studies suggest that modern formulations of PACs, such as hydrogels and nanoformulations, enable precise targeting of PACs to wounds, potentially significantly increasing treatment effectiveness. However, the existing data are insufficient to draw definitive conclusions about the optimal delivery systems for these compounds. Ultimately, it is essential to conduct thorough investigations into dermal toxicity, long-term safety, and pharmacokinetic factors, including bioavailability and tissue distribution, to ensure the safety and efficacy of PAC-containing products.

Implementing research strategies that address the mentioned limitations will facilitate broader adoption of PACs in dermatological and pharmaceutical applications.

## Figures and Tables

**Table 1:** The representative PACs-abundant plant species and organs.

Plant Material	Extract/Sample	Identified PACs	Identification Method	PACs Content	Quantitation Method	Ref.
*Annona cherimola* Mille; fruit, pulp, peel, seed	Methanol-water (4:1, *v*/*v*) extracts of pulp/peel/seed powder	A-type dimers; B-type dimers, trimers, tetramers	HPLC-DAD-QTOF-MS	38.30–46.80 mg/100 mg dw (pulp); 74.02–86.40 mg/100 mg dw (peel); 1.03–2.61 mg/100 mg dw (seed)	HPLC-DAD-QTOF-MS	[71]
*Annona crassiflora* Mart.; fruit peel	Ethyl acetate fraction of the ethanolic extract	B-type dimers, trimers, tetramers, pentamers	HPLC-ESI-MS/MS	758 ± 12.00 mg catechin equivalents (CE)/g	Vanillin assay	[72]
*Aronia arbutifolia* (L.) Pers.; *A. melanocarpa* (Michx.) Elliott; *A. mitschurinii* A.K. Skvortsov and Maitul; *A. prunifolia* (Marshall) Rehder; fruit	Acidified acetone 70% extract from lyophilized berry powder	B-type dimers, trimers, oligomers (4–6 units, 7–10 units), polymers > 10	HPLC-UV-MS	9.28 ± 4.49 to 12.20 ± 7.28 mg cyanidin equivalents (CYE)/g dw;	4-dimethylamino-cinnamaldehyde (DMAC) assay	[73]
				1.93 ± 0.14 to 2.17 ± 1.34 mg CYE/g dw	UHPLC-DAD-MS	
*A. melanocarpa* Michx. Elliott (various cultivars) *A. prunifolia* (Marshall) Rehder (cv. Aron); fruit	Ethanol-water (7:3, *v*/*v*) pulp extract from frozen berries	B-type dimers (B2, B5), trimers (C1)	HPLC-DAD-FLD	1396 ± 24.00 to 2524 ± 37.70 mg procyanidin B2 equivalents (PB2E)/100 g fw	Bate–Smith assay	[74]
*Cinnamomum cassia* (L.) J.Presl; *C. burmanni* (Nees & T.Nees) Blume, *C. loureiroi*, *C. verum* J. Presl; bark	Methanol-water extract (6:4, *v*/*v*) from powdered cinnamon bark	A-type dimers, trimers, tetramers, pentamers; B-type dimers, trimers, tetramers, pentamers	UHPLC-HRMS	No available data	-	[52]
*Cinnamomum zeylanicum* (various cultivars); bark	Various extracts from bark (the highest content of PACs for decoction water extract)	No available data	-	1.00 ± 0.01 to 40.10 ± 0.10 mg CE/g dw	Vanillin assay	[75]
*Cotoneaster bullatus* Bois; *C. divaricatus* Rehder and E.H.Wilson; *C. dielsianus* E. Pritz.; *C. horizontalis* Decne; *C. hjelmqvistii* Flinck et B. Hylmö; *C. integerrimus* Medik.; *C. lucidus* Schltdl.; *C. melanocarpus* Lodd. ex C.K. Schneid.; *C. nanshan* Mottet; *C. tomentosus* Lindl.; *C. splendens* Flinck et B. Hylmö; *C. zabelii* C.K. Schneid; leaf	Defatted methanol-water (7:3, *v*/*v*) leaf extracts	B-type dimers, trimers (C1), tetramers	UHPLC-PDA-ESI-QTOF-MS	2.14 ± 0.03 to 15.00 ± 0.08% CYE (*C. melanocarpus* and *C. bullatus*, respectively)	Butanol/HCl assay	[54]
*Crataegus laevigata* (Poir.) DC., *C. monogyna* Jacq.; leaf, fruit	Ethanol-water (2:1, *v*/*v*) extracts from leaves and fruits	A-type dimer; B-type dimers and trimers	UPLC-ESI-Q-TOF-MS/MS	No available data	-	[76]
*Gaultheria procumbens* L.; leaf	Various extracts, including methanol-water (75:25, *v*/*v*) and ethyl acetate leaf extracts	A-type dimers, trimers, B-type dimers (B1, B2, C1), trimers	HPLC-PDA-ESI-MS^3^;	36.90 ± 0.35 to 175 ± 2.35 mg CYE/g dw (ethyl acetate and methanol-water extracts, respectively)	Butanol/HCl assay	[77]
				9.67 ± 0.16 to 64.00 ± 2.01 mg CYE/g dw (ethyl acetate and methanol-water extracts, respectively)	HPLC-PDA	
*Litchi chinensis* Sonn. (cv. Hemaoli); fruit	Methanol-water (7:3, *v*/*v*) extract from pulp;	A-type dimers (A1, A2), trimers, tetramers; B-type dimers (B1, B2), trimers (C1), tetramers	LC-ESI-Q-TOF-MS; ESI-MS; NMR Spectroscopy	12.10 ± 0.02 mg PB2E/g dw	DMAC assay	[78]
*Malus domestica* Borkh.; fruit	Acetone-water (7:3, *v*/*v*) extract from freeze-dried fruits	A-type dimers, trimers B-type dimers, trimers, tetramers, pentamers	UPLC-DAD-ESI-TQ-MS	No available data	-	[79]
*Malus pumila* Mill.; fruit	Methyl acetate fraction of extract from freeze-dried fruits	B-type dimers (B1, B2), trimers (C1), tetramers, oligomers (5–10 units)	HPLC-ESI/MS; MALDI-TOF/MS	No available data	-	[80]
*Prunus spinosa* L.; flower	Defatted methanol-water (7:3, *v*/*v*) flower extract and its various fractions	A-type dimers	UHPLC-PDA-ESI-MS^3^	45.10 ± 2.38 mg CYE/g dw (extract) 12.40 ± 0.25 mg CYE/g dw to 109.40 ± 3.71 mg CYE/g dw (water residue and ethyl acetate fraction, respectively)	Butanol/HCl assay	[81]
*Sorbus domestica* L.; leaf	Defatted methanol-water (7:3, *v*/*v*) leaf extract and its various fractions (the highest PAC content for n-butanol and ethyl acetate fractions)	B-type dimers, trimers (C1)	UHPLC-PDA-ESI-MS^3^	19.20 ± 0.90 to 183 ± 2.40 mg CYE/g dw	Butanol/HCl assay	[82]
*Theobroma cacao* L. (various cultivars); seed	Acidified methanolic extract from cocoa powder	B-type dimers (B2)	UHPLC-DAD-ESI-HR-MS^n^	12.70 ± 0.11 to 25.50 ± 0.12 mg/100 g dw (procyanidin B2)	UHPLC-DAD-ESI-MS^n^ after alkaline hydrolysis	[83]
*Theobroma cacao* L.; seed	Acidified methanolic extract from defatted cocoa powder	No available data	-	16.10 ± 2.96 to 27.30 ± 0.78 g CE/100 g dw	Vanillin assay	[84]
*Vaccinium angustifolium* Aiton; *V. macrocarpon* Aiton; *V. uliginosum* L.; *V. vitis idaea* L.; fruit	Acidified methanol-water (7:3, *v*/*v*) extracts from freeze-dried fruits;	A- and B-type dimers, trimers, tetramers, pentamers, heptamers, polymers > 8 units	HPLC-FLD-DAD;	27.30 ± 3.60 to 228 ± 4.20 mg CYE/g dw	HPLC-PDA;	[85]
*Vicia faba* L.; seed	Acetone-water extract (7:3, *v*/*v*) from seed coats	A-type dimer B-type dimers (B1, B2, B3) and trimer (C1)	ESI-MS	No available data	-	[86]
*Vitis vinifera* L.; seed	Aqueous extract from seeds	A-type dimers, trimers, tetramers, pentamers; B-type dimers (B2), trimers (C1), tetramers (D1), oligomers (5–13-mers)	LC-Chip/ESI-Q-TOF–MS	No available data	-	[87]
*Vitis vinifera* L.; fruit, fruit skin, seed	Methanol-water-formic acid (50:48.5:1.5, *v*/*v*) from whole fruit, skin and seeds	B-type dimers (B1, B2, B4), other dimers and oligomers (no additional data)	HPLC-MS-MRM;	7.79 ± 1.07 to 12.9 ± 0.70 mg procyanidin B1 equivalents/kg grape fw (dimers; skin); 214 ± 9.45 to 333 ± 35.70 mg CE/kg grape fw (oligomers; skin); 199 ± 26.90 to 277 ± 16.30 mg procyanidin B1 equivalents/kg grape fw (dimers; seed) 0.82 ± 0.13 to 1.03 ± 0.10 mg CE/kg grape fw (oligomers; skin)	HPLC-MS-MRM	[88]
*Vitis vinifera* L. (various cultivars); fruit	Methanol-water (2:1, *v*/*v*) extract from fruits	B-type dimers (B1, B2, B3, B4, B5), trimers (T2, T3, T4, C1)	UHPLC/QTOF-MS	5.40 to 20.60 mg/kg grapes	UHPLC/QTOF-MS	[89]

## Data Availability

No new data were created or analyzed in this study. Data sharing is not applicable to this article.

## Supplementary Materials

The following supporting information can be downloaded at https://www.mdpi.com/article/10.3390/ijms262010116/s1.

## Abbreviations

The following abbreviations are used in this manuscript:

AMPs	Antimicrobial peptides
A549	Human non-small-cell lung carcinoma cell line
CAT	Catalase
CE	Catechin equivalents
COX-2	Cyclooxygenase-2
CYE	Cyanidin equivalents
DEX	Dexamethasone
DMAC	4-dimethylamino-cinnamaldehyde
ECM	Extracellular matrix
EMPB	*Mallotus philippinensis* bark extract
EPCs	Endothelial progenitor cells
FBS	Fetal bovine serum
FGF	Fibroblast growth factor
FLG	Filaggrin
GM-CSF	Granulocyte-macrophage colony-stimulating factor
GSH	Glutathione
GSH-Px	Glutathione peroxidase
GSP	Grape seed extract, a commercially available product containing 95% dw PACs
GSPE	Grape seed extract, a commercially available product containing 54% dimeric, 13% trimeric, and 7% tetrameric PACs
GSSG	Glutathione disulfide
HaCaT	Immortalized human keratinocyte cell line
HAEC	Human aortic endothelial cells
HDFa	Human dermal fibroblasts
HFF	Human foreskin fibroblasts
HG	High-glucose
HO-1	Heme oxygenase-1
Hs27	Human dermal fibroblasts
HUVECs	Human umbilical vein endothelial cells
IgE	Immunoglobulin E
IL	Interleukin
IFN-γ	Interferon gamma
IMQ	Imiquimod
iNOS	Inducible nitric oxide synthase
JNK	c-Jun N-terminal kinase
KUM6	Mouse mesenchymal stem cells
L929	Immortalized murine fibroblast cell line
LTB4	Leukotriene B4
MAPK	Mitogen-activated protein kinase
NHEKs	Normal human epidermal keratinocytes
NF-κB	Nuclear factor κB
Nrf2	Nuclear factor erythroid 2-related factor 2
NIH-3T3	Normal immortalized mouse embryonic fibroblasts
NIR	Near-infrared light
OPCs	Oligomeric procyanidins
OPCG	Oligomeric procyanidins from grape seeds
PAC(s)	Proanthocyanidin(s)
PB2	Procyanidin B2
PB3	Procyanidin B3
PB2E	Procyanidin B2 equivalents
PBO/PBOF	Hydrogel composed of polyvinyl alcohol (P), borax (B), oligomeric procyanidins (O), with or without ferric ion (F)
PGE_2_	Prostaglandin E_2_
PGES-1	Microsomal prostaglandin E synthase-1
pNHEK	Primary human epidermal keratinocytes
pNHD	Primary human dermal fibroblasts
PDGF	Platelet-derived growth factor
ProNPS	Bimetallic gold-silver nanoparticles modified with procyanidin B2
RAW264.7	Transformed murine macrophages
RRP	B-type catechin octamer from red-kerneled rice
ROS	Reactive oxygen species
SOD	Superoxide dismutase
TF	Tissue factor
TGF-β	Transforming growth factor beta
Th	T-helper cells
TNF-α	Tumor necrosis factor alpha
TSLP	Thymic stromal lymphopoietin
UV	Ultraviolet radiation
VEGF	Vascular endothelial growth factor
3-NT	3-nitrotyrosine
3T3-L1	Immortalized mouse fibroblasts
4-HNE	4-hydroxynonenal
5-LOX	5-lipoxygenase
