# Supplementary material for: Proanthocyanidins as Therapeutic Agents in Inflammation-Related Skin Disorders
Source: Int J Mol Sci. 2025 Oct 17;26(20):10116. doi: 10.3390/ijms262010116 (PMC12564351; doi:10.3390/ijms262010116)
Supplement: Supplementary file 1 [file ijms-26-10116-s001.zip › ijms-3899801-supplementary.pdf]

Supplementary Material for:

Proanthocyanidins as Therapeutic Agents in Inflammation-Related Skin Disorders

Aleksandra Prokop [1], Anna Magiera [1] and Monika Anna Olszewska [1,*]

[1] Department of Pharmacognosy, Faculty of Pharmacy, Medical University of Lodz, Muszynskiego 1, 90-151 Lodz, Poland; aleksandra.prokop@umed.lodz.pl (A.P.); anna.magiera@umed.lodz.pl (A.M.); monika.olszewska@umed.lodz.pl (M.A.O.)

* Correspondence: monika.olszewska@umed.lodz.pl; Tel.: +48-426779172

Supplementary Materials

Table S1. Wound-healing activity of proanthocyanidins (PACs) – in vitro, ex vivo, and in vivo studies.

Table S2. Anti-inflammatory activity of proanthocyanidins (PACs) – in vitro and in vivo studies.

Table S3. Photoprotective activity of proanthocyanidins (PACs) – in vitro studies.

Abbreviations

References

Table S1. Wound-healing activity of proanthocyanidins (PACs) - in vitro, ex vivo, and in vivo studies.

Study type	Tested substances	Dosage	Objective	Model	Control	Tested parameters	Observed effects	Ref.
In vitro	Leucoselect® - a commercially available, standardized extract of grape seed (*Vitis vinifera* L.) containing 95.0-105.0% dw of PACs and 13.0-19.0% dw of catechin and epicatechin;	Proliferation tests: 0.032–0.5 mg/mL (MTT assay); 0.125–1.0 mg/mL (Neutral Red uptake assay);<br><br>Oxidative stress test: 1.0 mg/mL.	The effect of pretreatment with PACs on fibroblast proliferation (with or without photo-irradiation) and intracellular H_2O_2-induced oxidative stress.	Immortalized mouse fibroblasts (3T3-L1).	For proliferative response tests: Negative control: Cells treated with physiological saline with or without photo-irradiation.<br><br>For oxidative stress test: Negative control: Cells treated with physiological saline with or without H_2O_2 induction.<br><br>No positive controls.	Cell proliferation; Intracellular H_2O_2-induced oxidative stress level - expressed as 2′,7′-dichlorodihydrofluorescein (DCF) equivalent; Cellular incorporation of PACs.	↑ fibroblasts proliferation in MTT assay by PACs pretreatment (for 1 min.) at dose-dependent manner (regardless of photo-irradiation, $p < 0.01$),<br>Percentage increase in the viable cells in treatment groups *vs*. control:<br>-without photo-irradiation:<br>at 0.125 mg/mL – about 25%,<br>at 0.25 mg/mL – about 50%,<br>at 0.5 mg/mL – about 75%,<br>-with photo-irradiation:<br>at 0.125 mg/mL – about 20%,<br>at 0.25 mg/mL – about 60%,<br>at 0.5 mg/mL – nearly 100%,<br>↑ fibroblasts proliferation in NR uptake assay by PACs pretreatment (for 1 min.) at dose-dependent manner ($p < 0.01$),<br>Percentage increase in the viable cells in treatment groups *vs*. control:<br>-only without photo-irradiation:<br>at 0.125 mg/mL – about 60%,<br>at 0.25 mg/mL – about 75%,<br>at 0.5 mg/mL – about 85%,<br>at 1.0 mg/mL – about 110%,<br>↑ fibroblasts proliferation in MTT assay by PACs pretreatment (for 0.5-32 min.) at dose-dependent manner ($p < 0.01$),<br>Percentage increase in the viable cells in treatment groups *vs*. control only without photo-irradiation:<br>at 0.5 mg/mL for 0.5 min.– about 50%,<br>at 0.5 mg/mL for 32 min.– about 55%,<br>↓ H_2O_2-induced intracellular oxidative stress: the DCF equivalent level decreased approximately 3-fold after PACs pretreatment ($p < 0.05$);<br>Incorporation of PACs into the cells in a short time (ca. 1 min.).	[1]

In vitro	Procyanidins B2, B5, C1, and D1 (PB2, PB5, PC1, PD1) from ethyl acetate fraction of *Combretum mucronatum* leaf ethanol-water (1:1) extract.<br><br>*Combretum mucronatum* leaves aqueous extract (with tannin content 13.0±1.3% w/w) with the same UHPLC profile as 50% ethanol-water extract.	Aqueous extract: 0.01-100 μg/mL;<br><br>PB2: 0.1-172 μM;<br>PB5, PC1, PD1: no available data on the concentrations tested.	The effect of the extract and procyanidins on skin cells viability, mitochondrial energy status, differentiation, proliferation and migration.	Immortalized human keratinocyte cell line (HaCaT);<br><br>Primary human epidermal keratinocytes (pNHEK);<br><br>Primary human dermal fibroblasts (pNHD).	Negative control: untreated cells;<br><br>For cellular differentiation: Positive control: cells treated with $CaCl_2$;<br><br>For cell viability: Positive control: 10% fetal calf serum (FCS) medium.	Cell viability; Cytotoxicity; Cellular energy status (MTT); Cell proliferation; Cell differentiation and differentiation-specific markers (involucrin and cytokeratin K10); Cell migration; Collagen production.	For aqueous extract:<br>Inhibition of HaCaT keratinocytes proliferation at 50 and 100 μg/mL with no necrotic activity ($p < 0,001$);<br>No significant influence on energy status of HaCaT cells;<br>↑ expression of involucrin and cytokeratin K10 (differentiation markers) in pNHEK at 1.0 and 10.0 μg/mL during long-term treatment (7 days);<br>↑ expression of cytokeratin K10 in pNHEK at 0.01 to 1.0 μg/mL during short-term treatment (24 h), no effect for involucrin;<br><br>For PB2:<br>↑ expression of involucrin and cytokeratin K10 in pNHEK during long-term treatment at 1.0 and 10.0 μM;<br>Inhibition of HaCaT proliferation at ≥ 10 μM with no necrotic activity ($p < 0,001$);<br><br>For PB5, PC1, and PD1:<br>No significant effect on expression of involucrin and cytokeratin K10 in pNHEK.<br><br>For aqueous extract and PB2, PB5, PC1, and PD1:<br>No significant effects on pNHDF fibroblasts migration;<br>No significant effect on collagen production.	[2]
In vitro	Grape seed PACs (GSP) - a commercially available, standardized grape seed extract (*Vitis vinifera* L.). PACs: 95% dw; total polyphenols: 90%-95% dw.	1.0-8.0 μg/mL	The GSP effect on endothelial cell protection and angiogenesis-related mechanisms under H_2O_2-induced oxidative stress condition.	Human umbilical vein endothelial cells (HUVECs) under H_2O_2-induced oxidative stress condition.	Negative control: Untreated cells under normal condition;<br><br>No positive control.	Cell viability; Expression of proteins: PINK1 and Parkin (mitochondrial damage), P62 and LC3 (autophagy); Reactive Oxygen Species (ROS) levels; Activity of antioxidant enzymes: catalase (CAT) and manganese superoxide dismutase	No significant toxic effect for GSP at 1.0, 2.0 ,4.0 μg/mL (optimal concentration 4.0 μg/mL, GSP at 4.0 μg/mL used for further tests);<br>↓ mitochondrial autophagy and mitochondrial damage: ↓ expression of PINK1, Parkin (almost 2-fold lower in treatment group *vs.* H_2O_2-group) and LC3-II ($p < 0.05$);<br>↓ mitochondrial autophagy: ↑ expression of P62 (above 2-fold higher than in H_2O_2-group) ($p < 0.05$);<br>↓ ROS levels by lower DHE staining ($p < 0.01$);<br>↑ activity of MnSOD by 1.3-fold and CAT by 3-fold ($p < 0.05$) *vs.* wound group;<br>↑ expression of p-JNK (almost 2- fold higher effect *vs.* H_2O_2-group) and FOXO3a (about 1.5-fold higher effect *vs.* H_2O_2-group) and activation of the p-JNK/FOXO3a signaling pathway ($p < 0.05$).	[3]

						(MnSOD);<br>Expression of p-JNK/FOXO3a.		
In vitro	Procyanidin B2 (PB2), 95% purity.	For endothelial progenitor cells (EPCs) functionality tests: 0.1, 0.5, 2.5 μmol/L<br><br>For further tests:<br>2.5 μmol/L	Protective effect of PB2 on the function of endothelial progenitor cells under under high glucose (HG)-mimicked diabetic hyperglycaemic condition.	Human umbilical cord blood endothelial cells (EPCs) treated with high-glucose (HG).	Negative control:<br>Cells treated with mannitol without HG;<br><br>No positive control.	EPCs functionality: angiogenesis (tube formation, tube length),<br>migration (wound closure rate and migration distance),<br>apoptosis (number of apoptotic cells);<br>ROS levels;<br>Oxidative damage markers levels: 3-nitrotyrosine (3-NT) and 4-hydroxynonenal (4-HNE);<br>Nrf signalling pathway activation;<br>mRNA and protein expression of Nrf and downstream genes (CAT and NQO-1).	↑ tube formation and relative tube length in a dose-dependent manner, at the similar level to mannitol without HG ($p < 0.05$);<br>↓ HG-induced apoptosis of EPCs in a dose-dependent manner – about 2.5-fold lower cell apoptosis after treatment by PB2 at 2.5 μmol/L *vs*. HG-group ($p < 0.05$);<br>↑ wound closure rate and the migration distance EPCs in a dose-dependent manner, increase in wound healing rate about 40% by PB2 at 5 μmol/L *vs*. HG-group ($p < 0.05$);<br>↓ ROS level (DHE staining) – almost 2.5-fold lower by PB2 at 2.5 μmol/L *vs*. HG-group ($p < 0.01$);<br>↓3-NT level about 2-fold lower *vs*. HG-group ($p < 0.01$) and ↓ 4-HNE level about 3-fold lower *vs*. HG-group ($p < 0.01$);<br>↑ mRNA expression of:<br>Nrf about 2-fold higher *vs*. HG-group ($p < 0.01$);<br>CAT about 2-fold higher *vs*. HG-group ($p < 0.05$);<br>NQO-1 about 4-fold higher *vs*. HG-group ($p < 0.01$);<br>↑ protein expression of:<br>Nrf about 2-fold higher *vs*. HG-group ($p < 0.01$);<br>CAT about 2-fold higher *vs*. HG-group ($p < 0.01$);<br>NQO-1 about 2-fold higher *vs*. HG-group ($p < 0.05$).	[4]
In vitro	Grape seed PACs extract (GSPE), a commercially available product containing 54% dimeric, 13% trimeric, and 7% tetrameric PACs	2.5, 5.0, 10.0 μg/mL.	The effect of GSPE on wound healing by regulation of VEGF level under oxidative and pro-inflammatory condition.	Immortalized human keratinocyte cell line (HaCaT) with or without oxidative stress (induced by H_2O_2) or with or without inflammation	Negative control:<br>Untreated cells;<br><br>No positive control.	mRNA and protein expression of VEGF;	↑ mRNA expression of VEGF – additional intensification in response to H_2O_2, additional sensitisation of cells to oxidative stress (no data of statistical significance);<br>↑ protein expression of VEGF by GSPE under H_2O_2 condition in a dose-dependent manner ($p < 0.05$);<br>↑ protein expression of VEGF by GSPE under TNF-*α* stimulation – increase by above 10-fold by GSPE at 10 μg/mL *vs*. TNF-*α*-stimulated cells without GSPE ($p < 0.05$).	[5], [6]

				(induced by TNF-α).				
In vitro	PACs-rich fractions of crude extracts from various Alaskan berries: Bog blueberry (*Vaccinium uliginosum* L.), BF;<br>Crowberry (*Empetrum nigrum*), CF;<br>Lingonberry (*Vaccinium vitis-idaea* L.), LF;<br><br>BF:<br>22.0% PACs, with 10.4% A-type dimers and 6.09% B-type dimers;<br>CF: 34.0% PACs, with 10.0% A-type dimers, and 9.03% B-type dimers;<br>LF: 52% PACs, with 4.8% A-type dimers and 12.3% B-type dimers;<br><br>PB2	For anti-inflammatory and antioxidant and migration tests:<br>50.0 μg/mL;<br><br>For mitochondrial activity tests of PB2: 10.0 μM	Wound-healing and anti-inflammatory activities of extracts and PACs.	LPS-stimulated RAW 264.7 macrophages.<br><br>Human dermal fibroblasts (HDFa);	For anti-inflammatory and antioxidant tests:<br>Blank control: untreated cells,<br>Negative control: vehicle,<br>Positive control: dexamethasone (DEX) at 10 μM<br><br>For fibroblasts migration:<br>Negative control: vehicle (0.5% ethanol)<br>Positive control: 10% fetal bovine serum (FBS)	ROS and NO levels;<br>mRNA expression of COX-2 and iNOS (Inducible nitric oxide synthase);<br>Fibroblasts migration;<br>Mitochondrial activity (oxidative phosphorylation, glycolysis).	↓ ROS level by all fractions, reduction by about 38% *vs.* 45% for DEX ($p < 0.01$);<br>↓ NO level by all fractions, the highest reduction after treatment with LF by above 55% *vs.* 80% for DEX ($p < 0.001$);<br>↓ COX-2 expression by all fractions, strongest effect of BF by 50% *vs.* 60% for DEX ($p < 0.0001$);<br>↓ iNOS expression by all fractions, the highest reduction after treatment with CF and LF by above 55% *vs.* 65% for DEX ($p < 0.001$);<br>↑ fibroblast migration by all fractions (especially in early stages of wound healing process), the strongest effect for LF ($p < 0.001$);<br>Non-significant effect of PB2 on oxidative phosphorylation;<br>↑ glycolysis by PB2 ($p < 0.05$);	[7]
In vitro	PACs-rich fraction (PRF) from lingonberry (*Vaccinium vitis-idaea* L.) leaf acetone-water (4:1) extract;<br>PACs: A-type dimers (PA1, PA2, PA4), B-type dimers (PB1, PB2, PB3), B-type trimers (PC1);<br>Total PAC amount: 377± 2.9 mg/g dw,	Cytotoxicity assay: 3.9 to 125.0 μg/mL;<br><br>Cell migration: 5.0, 10.0, 20.0 μg/mL	Wound-healing potential of a PRF and its incorporation into polymeric films for optimized dermal delivery.	Human foreskin fibroblasts (HFF).	Negative control: Untreated cells.<br><br>No positive control.	Cell viability;<br>Cell migration;<br>Size of wound area;<br>Polymeric film properties: thickness, moisture, stickiness;<br>PAC release from films.	↑ fibroblasts migration by PRF at 5.0 μg/mL (1.6-fold higher effect than negative control);<br>↓ wound area by PRF at 5.0 μg/mL after 20 h of incubation (13.3 ± 4.1% *vs.* 21.8 ± 2.3% in negative control group, $p < 0.05$);<br>inhibition of fibroblasts migration by PRF at 20.0 μg/mL);<br>optimal film composition: 0.30 g methylcellulose, 0.05 g hydroxyethyl cellulose, and 3.0 g PEG 400;<br>Films exhibited regulated efficiency of PAC release (up to 56.5% over 4 hours) and preferable mechanical attributes (stickiness and moisture content).	[8]

	PA1 (principal compound): 50.6 ± 0.3 mg/g dw (51.1% of total PACs), PA2: 16.5 ± 0.5 mg/g dw (16.7% of total PACs).							
In vitro	Oligomeric PACs (OPCs) from Grape seed extract (*Vitis vinifera* L.).<br><br>*no specific data on composition	2.5-20.0 μg/μL.	OPC effects on fibroblasts' procollagen production relevant to wound healing and collagen production.	Human dermal fibroblasts (Hs27) exposed to TGF-β1 or ascorbic acid.	Negative control: untreated cells;<br><br>No positive control.	Cell viability; Procollagen type I expression and secretion; Intracellular localization of procollagen; mRNA expression of procollagen type I biosynthesis enzymes (HSP47, P4Ha, P4Hb);	No cytotoxicity;<br>Inhibition of procollagen secretion induced by TGF-β1 and promoting intracellular accumulation of procollagen after exposure to TGF- β1;<br>Procollagen secretion (by Western blot):<br>↓ procollagen secretion by OPCs in cells without TGF-β1 in a dose-dependent manner – complete inhibition after 24 h by OPCs at 10.0 and 20.0 μg/μL ($p < 0.01$), effect maintained for 48 h (non-significant, $p > 0.05$);<br>↓ procollagen secretion by OPCs at 10.0 μg/μL in cells exposed to TGF-β1 after 6 h ($p > 0.05$);<br>↓ procollagen secretion by OPCs at 10.0 μg/μL combined with TGF-β1, secretion returned to control level (non-significant, $p > 0.05$);<br>Procollagen secretion (by immunochemistry):<br>↓ procollagen secretion by OPCs at 2.5 μg/μL by above 2-fold and 0.38-fold at 5 μg/μl *vs*. group exposed to TGF-β1 (non-significant, $p > 0.05$);<br>↑ intracellular accumulation of procollagen in a dose-dependent manner ($p < 0.01$);<br>OPCs not change procollagen type I biosynthesis enzymes mRNA level;<br>↓ procollagen secretion by OPCs at 10.0 μg/μL alone *vs*. control and by OPCs at 10.0 μg/μL combined with ascorbic acid *vs*. group exposed to ascorbic acid ($p < 0.05$);<br>↓ intracellular accumulation of procollagen by OPCs at 10.0 μg/μL combined with ascorbic acid ($p < 0.05$).	[9]
In vitro	*Mallotus philippinensis* bark extract (EMPB) containing cinnamtannin B1 (31% of total EMPB composition);	0.16, 0.8, 4.0, 20.0 μg/mL	Effects of EMPB and cinnamtannin B1 on MSC on proliferation and migration;	Mouse mesenchymal stem cells (KUM6);	Negative control: untreated cells;	Cell migration and proliferation;	For KUM6 cells:<br>↑ cell proliferation by EMPB at 0.16-4.0 μg/mL, increase by 50 % at 4.0 μg/mL ($p < 0.01$),<br>EMPB was toxic at 20.0 μg/mL;<br>↑ cell migration by EMPB at 0.8 and 4.0 μg/mL EMPB by about 1.4-fold ($p < 0.05$);	[10]

	Cinnamtannin B1		Effects of EMPB on the proliferation and migration of keratinocytes, fibroblasts, macrophages, and vascular endothelial cells.	Normal immortalized mouse embryonic fibroblasts (NIH-3T3);<br><br>Normal human epidermal keratinocytes (NHEKs);<br><br>Transformed murine macrophages (RAW264.7);<br><br>Human aortic endothelial cells (HAEC).	Positive controls: For cell proliferation: 1% (FBS) in DMEM;<br><br>For cell migration: platelet-derived growth factor (PDGF) in DMEM;		↑ cell proliferation by cinnamtannin B1 at 4.0 and 20.0 μg/ml, increase by about 30-35% ($p < 0.01$);<br>↑ cell migration by cinnamtannin B1 at 0.8 μg/mL highest increase by 1.4-fold ($p < 0.01$);<br><br>For NIH-3T3 cells:<br>↑ cell proliferation by EMPB, highest increase by 60% at 20.0 μg/mL ($p < 0.01$);<br>↑ cell migration by EMPB at 0.8–20.0 μg/mL EMPB, increase by 3.4-fold at 20.0 μg/mL ($p < 0.01$);<br><br>For NHEK cells:<br>Cell proliferation – no significant effect;<br>↑ cell migration by EMPB at 4.0-20.0 μg/mL, increase by 1.6-fold at 4.0 μg/mL results are statistically significant ($p < 0.01$);<br><br>For RAW 264.7 cells:<br>↓ Cell proliferation at 20.0 μg/mL ($p < 0.01$);<br>Cell migration – no significant effect;<br><br>For HAEC:<br>Cell proliferation – no significant effect;<br>↑ cell migration by EMPB at 4.0-20.0 μg/mL, increase by 3-fold at 20.0 μg/mL ($p < 0.01$).	
In vitro	Bimetallic gold-silver nanoparticles Au@AgNPs modified with PB2 200 μM (Pro-NPS);<br><br>PB2	5.0 μg/mL of nanoparticles with 200 μM of PB2;<br><br>For PB2 alone: 5.0 μg/mL.	Effects of Au@AgNPs nanoparticles modified with PB2 and pure PB2 on keratinocyte differentiation, proliferation, epithelial integrity, and remodeling.	Immortalized human keratinocytes (HaCaT);	Negative control: Untreated cells;<br><br>Comparator group: unmodified Au@AgNPs nanoparticles (NPS).	Cell migration;<br>Expression of PCNA (proliferating cell nuclear antigen), E-cadherin (epithelial integrity), involucrin (differentiation), MMP-9 (matrix metalloproteinase, remodeling).	↑ keratinocytes migration by all tested substance: modified Au@AgNPs nanoparticles with 200 μL PB2 > unmodified Au@AgNPs nanoparticles > PB2 ($p < 0.01$);<br>↑ expression of PCNA, E-cadherin, MMP-9 by modified Au@AgNPs and by PB2 ($p \leq 0.05$);<br>No significant changes in the expression of involucrin.	[12]
In vitro	Oligomeric PACs from grape seeds (OPCs,	OPCs 10.0 mg/mL	Effects of hydrogels with	Immortalized murine	Negative control: gauze	Scavenging of ROS:	Scavenging ratios of PBO, $PBOF_{0.2}$, $PBOF_{0.6}$ and $PBOF_{1}$ hydrogels ($p < 0.0001$) towards:	[13]

	containing 1-5% PB2) incorporated into hydrogels PBO/PBOF with polyvinyl alcohol (P), borax (B), OPCs (O), with or without ferric ion from $FeCl_3$ (F).	*depending on the concentration of $FeCl_3$, the following hydrogels were tested<br>PBO (no $FeCl_3$)<br>$PBOF_{0.2}$ (0.2 mg/mL),<br>$PBOF_{0.6}$ (0.6 mg/mL) and<br>$PBOF_1$ (1.0 mg/mL).	incorporated OPCs: antioxidant, antibacterial and hemostatic.	fibroblast cell line (L929).	(coagulation tests);<br>Deionized water (antioxidant tests);<br>Liquid medium (antibacterial tests).<br><br>No positive control.<br><br>For wound healing in a *S. aureus*-infected nape skin wound model:<br>Negative control:<br>no treatment	hydroxyl radical ($HO^•$),<br>$ABTS^+$ radicalcation, nitric oxide ($NO^•$), superoxide anion ($O_2^{•-}$);<br>Intracellular ROS level;<br>Photothermal effect and stability;<br>Near-infrared (NIR) light antibacterial activity against *Staphylococcus aureus, Escherichia coli;*<br>Hemostatic activity;<br>Coagulation with calcified blood.	- $O_2^{•-}$: 84.0%, 78.5%, 86.5% and 85.1%;<br>- $NO^•$: 93.4%, 90.6%, 83.8%, 82.7%;<br>- $HO^•$: 63.0%, 48.2%, 33.7%, 9.9%;<br>↓ intracellular ROS levels by PBO and $PBOF_{06}$;<br>Dose-dependent and Fe^{3+}-dependent photothermal response and stability under NIR light (potential as light-triggered therapy);<br>Bacterial killing ratio of PBO, $PBOF_{0.2}$, $PBOF_{0.6}$ and $PBOF_{1.0}$ hydrogels:<br>against *E. coli*: 99.1%, 96.1%, 92.5%, 86.1%;<br>against S. aureus: 99.4%, 97.9%, 96.5%, 89.3%;<br>$PBOF_{0.6}$ hydrogel with 10 min NIR kills nearly 100% of bacteria (both *E. coli* and *S. aureus*);<br>↓ BCI (blood clotting index) by all hydrogels, lower than gauze ($p <$ 0.0001), suggesting all hydrogel possessed good hemostasis capability.	
In vivo	Grape seed PACs-enriched extract (GSPE) containing 54% dimeric, 13% trimeric, and 7% tetrameric PACs) commercially available product (from InterHealth Nutraceuticals)	2.5 mg in 25 μL (0.1 mg/μL), daily for 5 consecutive days, topically.	GSPE's effects on wound healing in a mouse excisional wound model.	Excisional wound model of male BalbC mice.	Negative control: Untreated mice;<br><br>No positive control.	Cell viability;<br>Wound closure rate (evaluation of wound area reduction on days 0, 3, 7, 10, and 14);<br>Histological analysis of wound area;<br>Oxidative signals promoting wound healing:<br>VEGF and tenascin expression and Glutathione disulfide (GSSG)/Glutathione (GSH) ratio;<br>4-HNE levels.	↓ wound area after GSPE treatment in time-dependent effect:<br>↓ wound area by about 40% after 1 day of treatment *vs*. control ($p <$ 0.05);<br>↓ wound area by about 80% after 11 days *vs*. control;<br>↑ oxidative signals promoting wound healing:<br>↑ VEGF and tenascin expression (especially at the wound edges) (p < 0.05);<br>↑ GSSG/GSH ratio about 27 % *vs*. control ($p < 0.05$);<br>↑ histological organization: improvement of epidermal and connective tissue formation;<br>maintaining GSH level;<br>↓ 4-HNE level (no data of statistical significance).	[5], [6]

In vivo	Bimetallic gold-silver nanoparticles Au@AgNPs modified with Procyanidin B2 200 μM (Pro-NPS);<br><br>PB2 purchased from commercial suppliers.	5.0 μg/mL of nanoparticles with 200 μL of Procyanidin B2<br><br>For Procyanidin B2 alone: 5 μg/mL	Effects of Au@AgNPs nanoparticles modified with Procyanidin B2 and Procyanidin B2 on wound healing, epithelial thickness, neutrophil infiltration and cytokine expression.	Excisional wound model of C57BL6 mice;	Negative control: Untreated mice.<br><br>No positive control.	Wound size; Epithelial thickness (reepithelialization); Number of neutrophils in wound area; Expression of TNF-α, PDGF-β, IL-1β, TGF-1β, VEGF.	↓ wound area: decrease (%) after 6 days: Control 40%, NPS 50%, Procyanidin B2 50%, Pro-NPS 75% ($p \leq 0.05$);<br>↑ epithelial thickness by Procyanidin B2 and Pro-NPS (non-significant, $p > 0.05$);<br>↑ number of neutrophils in wound area by Pro-NPS after 14 days ($p \leq 0.05$);<br>↑ expression of PDGF-β after 3 days of treatment of Procyanidin B2 ($p \leq 0.05$);<br>Non-significant changes in the expression of TNF-α, PDGF-β, IL-1β, TGF-1β, VEGF after treatment of Pro-NPS.	[12]
In vivo	*Mallotus philippinensis* bark extract (EMPB) containing cinnamtannin B1 (31% of total EMPB composition).	EMPB-I (0.4 μg/wound); EMPB-II (1.3 μg/wound); three times per week for 21 days, topically.	Effect of EMPB on wound healing, angiogenesis and reepithelialization in a diabetic mouse wound model.	Diabetic C57BLKS/Jlar-+ Leprdb/ + Leprdb mice	Negative control: PBS;<br><br>No positive control.	Wound size; Number of α-positive SMA capillaries; Granulation tissue area.	↓ wound size by EMPB-II from days 7 to 17; after 17 days percentage of the wound area after 17 days 42% *vs*. 55% in control group ($p < 0.05$; at 14th day $p < 0.01$).<br>↓ wound size (slight decrease) by EMPB-I from days 7 to 17, after 17 days percentage of the wound area after 17 days 52% *vs*. 55% in control group ($p < 0.05$; at 14th day $p < 0.01$).<br>↑ formation of granulation tissue by EMPB-II, percentage of the wound area on 14th day 68% *vs*. 58% in control group ($p < 0.05$);<br>↑ capillaries by EMPB-II – increase by above 2-fold *vs*. control ($p < 0.05$).	[10]
In vivo	Cinnamtannin B1	Cinnamtan-nin B1 I (1.2 μg/wound);<br><br>Cinnamtannin B1 II (2.4 μg/wound);<br><br>three times per week for 21 days, topically.	Effect of cinnamtannin B1 on wound healing, angiogenesis and reepithelialization in a diabetic mice wound model.	Diabetic mice C57BLKS/Jlar-+ $Lepr^{db}$/ + $Lepr^{db}$	Negative control: PBS;<br><br>No positive control.	Wound size; Histological analysis of epithelialization (on 14th day); Number of α-positive SMA capillaries; Granulation tissue area.	↓ wound size by cinnamtannin B1 II on day 5, 12, 14, 17, 19, and 21; percentage of wound area 10% lower *vs*. negative control ($p < 0,05$) on day 21;<br>↓ wound size by cinnamtannin B1 I on day 5, 19, and 21 ($p < 0,05$);<br>↑ epithelialization and formation of granulation tissue by cinnamtannin B1 II increased by 1.4-fold *vs*. negative control ($p < 0.01$), less potent effect for cinnamtannin B1 I;<br>↑ number of α-positive SMA capillaries by cinnamtannin B1 II by more than 3-fold *vs*. negative control ($p < 0,05$).	[11]

In vivo	Oligomeric PACs from grape seeds (OPCs, containing 1-5% PB2) incorporated into hydrogels PBO/PBOF with polyvinyl alcohol (P), borax (B), OPCs (O), with or without ferric ion from $FeCl_3$ (F).	OPCs: 10 mg/mL<br><br>Tested OPC-loaded hydrogels:<br>PBO (no $FeCl_3$)<br>$PBOF_{0.6}$ (0.6 mg/mL $FeCl_3$).	Effects of OPCs incorporated into hydrogels on hemostasis.	For hemostatic tests: model of bleeding from the femoral artery, tail amputation, or liver<br><br>For reepithelialization and collagen evaluation: Kunming mice.	For hemostatic tests:<br>Negative control:<br>no treatment;<br>Positive control: gauze;<br><br>For wound healing in a *S. aureus*-infected nape skin wound model:<br>Negative control:<br>no treatment.	Blood loss;<br>Hemostatic time;<br>Wound closure rate (on 10th day of treatment);<br>Histological assessment (on 10th day): re-epithelialization, dermal space length, collagen deposition;	↓mean amount of bleeding ($p < 0.0001$):<br>Percentage reduction of bleeding by hydrogel *vs*. gauze for specific models:<br>- femoral artery: 81.8% *vs*. 46.2%;<br>- tail: 80.4% *vs*. 27.7%;<br>- liver: 84.6% *vs*. 45.2%;<br>↓ hemostatic time ($p < 0.0001$):<br>Percentage reduction of hydrogel *vs*. gauze for specific models:<br>- femoral artery: 85.2% *vs*. 39.5%;<br>- tail: 91.0% *vs*. 37.2%;<br>- liver: 83.5% *vs*. 33.9%;<br>↑ wound closure ratio by all hydrogels ($p < 0.0001$):<br>$PBOF_{0.6}$ + NIR: 98.7%;<br>$PBOF_{0.6}$: 97.1%;<br>PBO: 95.6%;<br>Control: 80.6%;<br>↑ re-epithelialization:<br>complete in PBOF 0.6 and PBOF0.6 + NIR, when partial in PBO and control;<br>↓ Dermal space length: smallest in PBOF 0.6+NIR;<br>↑ collagen deposition: the most fibres and well-organized in $PBOF_{0.6}$ + NIR, moderate in $PBOF_{0.6}$.	[13]
Ex vivo	*Stryphnodendron adstringens* bark extract (SEG);<br>containing monomeric (epi)gallocatechins and their methylated and galloylated derivatives, oligomeric structures such robinetinidol-(epi)gallocatechin dimers;	170.0 mg of gel containing 1% of crude extract applied for 4, 7, 10, and 14 days, topically.	Effect of SEG on skin wound healing in rats with streptozotocin-induced (35 mg/kg) diabetes, effect on keratinocyte differentiation, proliferation, migration, and collagen maturation.	Streptozocin-induced diabetic wound in Wistar rats.	Negative control: base gel without SEG.<br><br>No positive control.	Wound histology: migration and cell differentiation;<br>Evaluation of length (after 4 and 7 days of treatment) and thickness (after 10 and 14 days) of the re-epithelialized surface;<br>Keratinocyte proliferation;<br>Number of cells in mitosis;	↑ length of re-epithelialized surface (↑ keratinocyte migration) after 4 days of treatment ($p < 0.05$);<br>Full thickness of re-epithelized surfaced reached at 10 and 14 days of treatment (↑ keratinocyte migration); better effect in the negative control at 14 days ($p < 0.05$);<br>↑ keratinocytes proliferation after 4 days ($p < 0.05$), however better results in negative control group after 10 days ($p < 0.05$);<br>↑ content of collagen type I fibers (mature collagen) in the wound at 4, 7, 10, 14 days than type III collagen fibers;<br>At 10 and 14 days, SEG stimulated ($p < 0.05$) formation of type I (mature) collagen better than negative control.	[14]

Total polyphenol content: 37.9 ± 1.54% dw.	Collagen type I and type III fibers analysis.

Table S2. Anti-inflammatory activity of proanthocyanidins (PACs) - in vitro and in vivo studies.

Study type	Tested substances	Dosage	Objective	Model	Control	Tested parameters	Observed effects	Ref.
In vitro	PACs – a commercially available product; no specific data of plant source, content and purity.	50, 75, 100 μg/mL; Safe levels for cellular studies: ≤ 100 μg/mL	Protective effects on psoriasis-like cell models induced by TNF-α.	Psoriasis-like keratinocytes (HaCaT) exposed to TNF-α.	Negative control: untreated cells;<br><br>Positive controls: cells pretreated with LY294002 (PI3K inhibitor) and ZnPP (HO-1 inhibitor).	IL-17 and IL-23 levels; JAK2/STAT3 pathway activation; PI3K/AKT pathway activation; HO-1 expression; SOD, CAT, GSH levels; ROS and MDA levels; Cell density; Keratinocyte proliferation.	↓ IL-17 by almost 1.3-fold in each concentration *vs.* negative control ($p < 0.01$);<br>↓ IL-23 levels in each concentration; the most potent effect at 75 μg/mL by almost 1.24-fold *vs.* negative control ($p < 0.01$);<br>↑ SOD in each concentration; the most potent effect at 75 μg/mL by 2-fold *vs.* negative control ($p < 0.01$);<br>↑ CAT in each concentration; the most potent effect at 100 μg/mL by 4.5-fold *vs.* negative control ($p < 0.01$);<br>↑ GSH in each concentration; the most potent effect at 50 μg/mL by 2-fold *vs.* negative control ($p < 0.01$);<br>↓ ROS in each concentration; the most potent effect at 75 μg/mL by 1.3-fold *vs.* negative control ($p < 0.01$);<br>↓ MDA in each concentration; the most potent effect at 75 μg/mL by 4.8-fold *vs.* negative control ($p < 0.01$);<br>↓ JAK2/STAT3 and PI3K/AKT expression ($p < 0.01$);<br>↑ HO-1 expression ($p < 0.01$);<br>For cells pretreated with ZnPP (PACs *vs.* ZnPP):<br>↓ keratinocite proliferation: 75 *vs.* 120 ($p < 0.01$);<br>↓ ROS: 5.5 *vs.* 8.5 ($p < 0.01$).<br>↓ MDA: 2 *vs.* 13 ($p < 0.01$);<br>↑ SOD: 27 *vs.* 9 ($p < 0.01$);<br>↑ CAT: 1.3 *vs.* 0.5 ($p < 0.01$);<br>↑ GSH: 60 *vs.* 25 ($p < 0.01$);<br>LY294002 (PI3K inhibitor) showed similar anti-inflammatory and antioxidant effects as PACs;<br>ZnPP (HO-1 inhibitor) in combination with PACs reversed the protective effects of PACs.	[15]
In vitro	PAC-enriched fraction from red-kerneled rice *Oryza sativa* L. (RRP); PACs: B-type catechin octamer, no data on purity	0-1000 μM for LOX inhibition 0-500 μM for COX inhibition.	The effects on lipoxygenases (LOX), prostaglandin E synthases (mPGES), and eicosanoid	Human/rat recombinant enzymes for non-cellular studies;	Negative control: untreated enzymes/cells;	Cytotoxicity; 5-LOX activity; Calcium chelation as mechanism of 5-LOX inhibition;	RRP IC_{50} (non-cellular tests):<br>human 5-LOX: 15.1 μM, mixed noncompetitive inhibition<br>rat 5-LOX: 7.0 μM, mixed noncompetitive inhibition, calcium ion chelation not involved in the inhibition mechanism<br>human 12-LOX: 39.0 μM,<br>human 15-LOX-2: > 100 μM	[16]

			production (LTB4, PGE2).	A549 cells (human non-small-cell lung carcinoma) for testing PGE2 secretion;<br><br>Rat basophilic leukemia (RBL-2H3) for testing LTB4 secretion.	Positive controls: Zileuton (5-LOX competitive inhibitor); EDTA (calcium chelating agent).	PGE2 and LTB4 production from intact cells; mPGES-1, COX-1, COX-2, 15-LOX-2, and 12-LOX activity; Inhibition kinetics.	COX-1, COX-2: no effects up to 500 μM<br>mPGES-1: 7.0 μM, noncompetitive inhibition<br>Catechin IC_{50}:<br>human 5-LOX: 2.2 μM, mixed noncompetitive inhibition<br>rat 5-LOX: 9.0 μM, mixed noncompetitive inhibition<br>human 15-LOX-2: 20.4 μM;<br>COX-1: 30 μM<br>mPGES-1: no effects up to 200 μM<br>Zileuton IC_{50}:<br>human 5-LOX: 0.86 μM, competitive inhibition<br>Cellular tests of RRP:<br>↓ LTB4 production in RBL-2H3 cells by ca. 40% at 1.0 μM<br>↓ PGE2 production in A549 cells by ca. 50% at 1.0 μM;	
In vitro	PACs-enriched aqueous extract of A*rachis hypogaea* L. (peanut) skin (PSE), no data on PAC content<br><br>PAC oligomers (11) isolated from PSE, e.g., PA1, PB3, and A-type trimers, including new epicatechin-(2β→*O*→7, 4β→6)-[epicatechin-(4β→8)]-catechin.	1 μM and 50 μM	Anti-inflammatory activity	LPS-stimulated human monocytic cell line (THP-1)	Negative control: untreated cells;<br><br>Positive control: DEX 10 μM.	Cytotoxicity; IL-6 and TNF-α secretion;	No cytotoxicity at 1-50 μM;<br>↓ IL-6, the strongest effect for epicatechin-(2β→*O*→7, 4β→6)-[epicatechin-(4β→8)]-catechin and epicatechin-(2β→*O*→7,4β →8)-*ent*-epicatechin at 50 μM, similar to that of DEX ($p < 0.05$);<br>↓TNF-α, the strongest effect for epicatechin-(2β→*O*→7, 4β→6)-[epicatechin-(4β→8)]-catechin at 50 μM, similar to that of DEX ($p < 0.05$);<br>Dimers and trimers had comparable inhibitory activity;<br>Tetramers had no significant effect, in some cases even directly enhanced cytokine production after LPS-stimulation.	[17]
In vivo	PAC-enriched fraction from red-kerneled rice *Oryza sativa* L. (RRP); PACs: B-type catechin octamer, no data on purity	2, 20, 200 mg/kg/day(applied topically for 5 days, 30 minutes before psoriasis-inducing imiquimod (IMQ) cream	Anti-inflammatory effects: inhibition of enzymes involved in psoriasis-like inflammation	Psoriasis-like skin lesions in ears of BALB/c mice, induced by IMQ	Negative control: mice treated with a vehicle (ethanol or DMSO)	Histological analysis: ear thickness, spinous layer thickness; Infiltrating neutrophils; Gene expression for 5-LOX, IL-17a, IL-22, and markers of keratinocyte differentiation or	↓ ear thickness by 2-fold at medium and highest dose ($p < 0.01$);<br>↓ spinous layer thickness by about 2-fold at medium and highest dose ($p < 0.01$);<br>↓ hyperplasia;<br>↓ neutrophil infiltration by 4-fold at medium and highest dose ($p < 0.01$);<br>↓ LTB4 level (reaching the normal level with the dose 2.5 mg/ear, equal to 200 mg/kg/day) ($p < 0.01$);<br>↓ expression of 12-HETE, IL-17, IL-22, S100a9, and Krt1b (in a dose dependent manner, $p < 0.01$).	[16]

						activation (S100a9 and Krt1); Eicosanoid production (LTB4, arachidonic acid, 12-HETE, PGD2, PGE2, PGF2α)		
In vivo	PACs isolated from the roots of *Rosa multiflora* Thunb.: PB3 and RM-1 (*ent*-guibourtinidol-(4β→6)-catechin)	100 mg of 1% PB3 or RM-1 cream applied topically to skin of atopic dermatitis (AD) mice for 4 weeks	Anti-inflammatory and immunomodulatory properties of PB3 or RM-1 in AD-like skin lesions.	AD-like skin lesions in NC/Nga mice	Negative control: mice treated with base cream;<br><br>Positive control: mice treated with 1% hydro-cortisone (HC) cream	The total skin severity score (sum of individual scores for skin dryness, erythema, excoriation, edema, erosion, hemorrhage, and scaling; Total serum eosinophil count; Total serum IgE level; Th2 cytokine (IL-4, IL5 and IL13) levels; Protein and mRNA expression for iNOS and COX-2	↓ severity of dryness, erythema, excoriation, edema, erosion, hemorrhage, scaling, significantly lower degree of clinical skin severity after PB3 and RM-1 treatment, effects comparable to HC ($p < 0.01$);<br>↓ eosinophil levels, effect of HC > RM-1 > PB3; reduction by 3-fold after RM-1 treatment ($p < 0.01$);<br>↓ IgE levels, effect of HC > RM-1 > PCB3; reduction by 4-fold after RM-1 treatment ($p < 0.01$);<br>↓ levels of IL-4, IL-5, and IL-13, reduction of IL-4 and IL-13 by PB3 and RM-1 comparable to HC ($p < 0.01$);<br>↓ expression of iNOS and COX-2 protein and the corresponding mRNA, effect of HC > RM-1 > PB3 ($p < 0.01$).	[18]

Table S3. Photoprotective activity of proanthocyanidins (PACs) – in vitro studies.

Study type	Tested substances	Dosage	Objective	Model	Control	Tested parameters	Observed effects	Ref.
In vitro	Oligomeric PAC-enriched fraction (DOP) from persimmon (*Diospyros kaki* Thumb.) fruit with a degree of polymerization of 3.1	5, 10 μg/mL	Photoprotective effects of DOP on oxidative stress, inflammation, and apoptosis in human keratinocytes exposed to UV-B radiation.	Immortalized human keratinocyte cell line (HaCaT), with or without UV-B irradiation.	Negative control: untreated cells (no UV-B irradiation);<br><br>No positive control.	Cell viability;<br>Cell apoptosis;<br>ROS levels;<br>MDA levels;<br>SOD levels;<br>GSH-Px (glutathione peroxidase);<br>GSH levels	↓ ROS levels in a dose-dependent manner, reduction by 50% at 10 μg/mL ($p < 0.01$);<br>↓ MDA levels in a dose-dependent manner, reduction by 1.5-fold at 10 μg/mL *vs*. negative control ($p < 0.01$);<br>↑ SOD levels in a dose-dependent manner, increase by 1.2-fold at 10 μg/mL *vs*. negative control ($p < 0.01$);<br>↑GSH-Px levels in a -dependent manner ($p > 0.01$);<br>↑ GSH levels in a dose-dependent manner, increase by 1.5-fold at 10 μg/mL *vs*. negative control ($p < 0.01$);<br>improvement of cell viability;<br>↓ apoptosis rate	[19]
In vitro	Nanoliposomes loaded with lotus (*Nelumbo nucifera* Gaertn.) seedpod oligomeric PACs (LPCN);<br>Non-encapsulated LPC<br><br>LPC: mixture of B-type monomers and dimers (no purity reported)	Melanin synthesis: 2 mg/mL<br><br>Cytotoxicity: 50, 100 μg/mL;<br><br>Cell viability: 12.5 and 25 μg/mL;<br><br>Oxidative stress and collagen loss: 12.5 μg/mL	Photoprotective efect of LPCN against UV-induced skin damage and melanogenesis	Human foreskin fibroblasts (HFF)	Negative control: non-treated and non-UV-irradiated cells;<br><br>Positive control: vitamin C at 12.5 μg/mL	Melanin synthesis: monophenolase and diphenolase activity;<br>SOD levels;<br>MDA levels;<br>Cell viability;<br>Hydroxyproline level (collagen loss)	↓ monophenolase activity: LPCN, $IC_{50} = 0.64 \pm 0.06$ mg/mL, reduction by 91.7% at 2 mg/mL; LPC, $IC_{50} = 0.56 \pm 0.03$ mg/mL;<br>↓ diphenolase activity: LPCN, $IC_{50} = 1.46 \pm 0.03$ mg/mL, reduction by 61.7% at 2 mg/mL; LPC, $IC_{50} = 0.73 \pm 0.02$ mg/mL, reduction by 94.8% at 2 mg/mL;<br>mild cytotoxicity for 100 μg/mL of LPCN and LPC;<br>strong protective effect on cell viability against UVB (order of efficacy: LPCN > LPC > vit. C);<br>↑ SOD level: effect of LPCN at 12.5 μg/mL > LPC at 12.5 μg/mL> vit. C ($p < 0.05$);<br>↓ MDA level: effect of LPN ($p > 0.05$) > LPCN at 12.5 μg/mL > vit. C ($p < 0.05$);<br>↑ hydroxyproline content (↓ collagen loss) in UVA injury model: effect of LPCN at 12.5 μg/mL > LPC at 12.5 > vit. C ($p < 0.05$).	[20]
In vitro	PACs-enriched extract (OW) and fractions F1-F3 of grape (*Vitis vinifera* L.) pomace	OW: 5, 10, 20 μg/mL;<br>F1-F3: 5 μg/mL;	Photoprotective effect of OW, and F1-F3 on skin cells against UV-induced oxidative damage	Immortalized human keratinocytes (HaCaT), with or without	Negative control: non-treated and non-UV-	ROS levels;	↓ baseline ROS levels in non-irradiated cells in a dose-dependent manner; fractions F1 and F2 at 5 μg/mL more effective than F3 (reduction 40-50% *vs*. reduction 20%);<br>↓ ROS levels after UVA and UVB exposure; similar efficacy (reduction by 50-60%) of F1-F2 at 5 μg/mL and OW at 10-20 μg/mL;	[21]

	containing PACs with different degrees of polymerization (mDP) and percentage of galloylation (% G):<br>OW (crude extract): mDP 1.7 and 15% G;<br>F1: mDP 2.7 and 25% G;<br>F2: mDP 3.7 and 31% G;<br>F3: non-galloylated monomers			UVB irradiation.	irradiated cells;		dose-dependent inhibition of p38 – fractions F1 and F2 at 5 μg/mL more effective than F3 (reduction by 50-60% *vs*. reduction by 25%), the strongest effect against UVB radiation;<br>strong inhibition of JNK1/2 – the strongest effect against UVB-irradiation, the most effective fraction: OW at 20 μg/mL (reduction by 60%)	
In vivo	Oligomeric PACs-enriched fraction (DOP) from persimmon (*Diospyros kaki* Thumb.) fruit with a degree of polymerization of 3.1 .	5 mg/cm^2 applied topically on the hairless skin in mice, 30 min before each UVB irradiation during 5 days	Photoprotective effect on skin cells against UV-induced oxidative damage	Skin of BalbC mice with or without UVB irradiation.	Negative control: Untreated mice;	Skin erythema;<br>Epidermal hyperplasia (epidermal thickness, number of epidermal cell layers);<br>MDA, SOD, GSH-Px, GSH levels;<br>Expression of p65 (NF-κB subunit);<br>IL-6 and TNF-α levels;<br>Cell apoptosis;<br>Expression of apoptosis markers: Bax, Bcl-2, P-53, Caspase 8, 9 and 3, MAPKs (p38, JNK, ERK);<br>Expression of Nrf2 and HO-1.	↓ epidermal thickness by 1.5-fold after P-OPC DOP treatment *vs*. UVB-irradiated group ($p < 0.05$);<br>↑ SOD, GSH levels ($p < 0.05$); ↑GSH-Px (non-significant);<br>↓ MDA levels by 1.5-fold after DOP treatment *vs*. UVB-group ($p < 0.01$);<br>↓ p65 phosphorylation by 40% after DOP treatment *vs*. UVB-group ($p < 0.05$);<br>↓ IL-6 by 25% after DOP treatment *vs*. UVB-irradiated group ($p < 0.05$);<br>↓ TNF-α by 25% after DOP treatment *vs*. UVB-irradiated group ($p < 0.05$);<br>↓ MAPKs levels;<br>↓ Bax expression ($p < 0.05$);<br>↑ Bcl-2 expression ($p < 0.01$);<br>↓ caspase 9, 3 ($p < 0.05$);<br>↑ Nrf, HO-1 expression	[19]

Abbreviations

The following abbreviations are used in this Supplementary Material:

A549	Human non-small-cell lung carcinoma cell line
BF	Bog blueberry (*Vaccinium uliginosum* L.) fraction
CAT	Catalase
CF	Crowberry (*Empetrum nigrum*) fraction
COX-2	Cyclooxygenase-2
DEX	Dexamethasone
DOP	Oligomeric PAC-enriched fraction from persimmon (*Diospyros kaki* Thumb.)
EMPB	*Mallotus philippinensis* bark extract
EPCs	Endothelial progenitor cells
FBS	Fetal bovine serum
GSH	Glutathione
GSH-Px	Glutathione peroxidase
GSP	Grape seed extract , a commercially available product containing 95% dw PACs
GSPE	Grape seed extract, a commercially available product containing 54% dimeric, 13% trimeric, and 7% tetrameric PACs
GSSG	Glutathione disulfide
HaCaT	Immortalized human keratinocyte cell line
HAEC	Human aortic endothelial cells
HC	Hydrocortisone
HDFa	Human dermal fibroblasts
HFF	Human foreskin fibroblasts
HG	High-glucose
HO-1	Heme oxygenase-1
Hs27	Human dermal fibroblasts
HUVECs	Human umbilical vein endothelial cells
IgE	Immunoglobulin E
IL	Interleukin
IMQ	Imiquimod
iNOS	Inducible nitric oxide synthase
JNK	c-Jun N-terminal kinase
KUM6	Mouse mesenchymal stem cells
L929	Immortalized murine fibroblast cell line
LF	Lingonberry (*Vaccinium vitis-idaea* L.) fraction
LPCN	Nanoliposomes loaded with lotus (*Nelumbo nucifera* Gaertn.) seedpod oligomeric proanthocyanidins
LTB4	Leukotriene B4
MAPK	Mitogen-activated protein kinase
MDA	Malondialdehyde
mDP	Degree of polymerization
MMP-9	Matrix metalloproteinase
MnSOD	Manganese superoxide dismutase
NHEKs	Normal human epidermal keratinocytes
NF-κB	Nuclear factor κB
Nrf2	Nuclear factor erythroid 2-related factor 2
NIH-3T3	Normal immortalized mouse embryonic fibroblasts
NIR	Near-infrared light
OPCs	Oligomeric procyanidins
OPCG	Oligomeric procyanidins from grape seeds
PACs	Proanthocyanidins

PA1/PA2/PA4	Procyanidin A1/A2/A4
PB1/PB2/PB3	Procyanidin B1/B2/B3
PBO/PBOF	Hydrogel composed of polyvinyl alcohol (P), borax (B), oligomeric procyanidins (O), with or without ferric ions (F)
PC1	Procyanidin C1 (B-type trimer)
PCNA	Proliferating cell nuclear antigen
PD1	Procyanidin D1 (B-type tetramer)
PGE_2	Prostaglandin E_2
PGES-1	Microsomal prostaglandin E synthase-1
PSE	PAC-enriched aqueous extract of A*rachis hypogaea* L. (peanut) skin
pNHEK	Primary human epidermal keratinocytes
pNHD	Primary human dermal fibroblasts
ProNPS	Bimetallic gold-silver nanoparticles modified with procyanidin B2
RAW264. 7	Transformed murine macrophages
RBL-2H3	Rat basophilic leukemia
RRP	Red-kerneled rice proanthocyanidin
RM-1	*ent*-guibourtinidol-(4β→6)-catechin
ROS	Reactive oxygen species
SOD	Superoxide dismutase
TGF-β	Transforming growth factor beta
TNF-α	Tumor necrosis factor alpha
UV	Ultraviolet radiation
VEGF	Vascular endothelial growth factor
3-NT	3-nitrotyrosine
3T3-L1	Immortalized mouse fibroblasts
4-HNE	4-hydroxynonenal
5-LOX	5-lipoxygenase

References

1. Tsuruya, M.; Niwano, Y.; Nakamura, K.; Kanno, T.; Nakashima, T.; Egusa, H.; Sasaki, K. Acceleration of Proliferative Response of Mouse Fibroblasts by Short-Time Pretreatment with Polyphenols. *Appl Biochem Biotechnol* **2014**, *174*, 2223–2235.
2. Kisseih, E.; Lechtenberg, M.; Petereit, F.; Sendker, J.; Zacharski, D.; Brandt, S.; Agyare, C.; Hensel, A. Phytochemical Characterization and in Vitro Wound Healing Activity of Leaf Extracts from *Combretum Mucronatum* Schum. & Thonn.: Oligomeric Procyanidins as Strong Inductors of Cellular Differentiation. *J Ethnopharmacol* **2015**, *174*, 628–636.
3. Chen, L.; Hao, L.; Yanshuo, C.; FangFang, W.; Daqin, C.; Weidong, X.; Jian, X.; Shaodong, C.; Hongyu, Z.; Ke, X. Grape Seed Proanthocyanidins Regulate Mitophagy of Endothelial Cells and Promote Wound Healing in Mice through P-JNK/FOXO3a/ROS Signal Pathway. *Arch Biochem Biophys* **2023**, *749*, 109790.
4. Fan, J.; Liu, H.; Wang, J.; Zeng, J.; Tan, Y.; Wang, Y.; Yu, X.; Li, W.; Wang, P.; Yang, Z.; et al. Procyanidin B2 Improves Endothelial Progenitor Cell Function and Promotes Wound Healing in Diabetic Mice via Activating Nrf2. *J Cell Mol Med* **2021**, *25*, 652–665.
5. Khanna, S.; Roy, S.; Bagchi, D.; Bagchi, M.; Sen, C.K. Upregulation of oxidant-induced VEGF expression in cultured keratinocytes by a grape seed proanthocyanidin extract. *Free Radic Biol Med.* **2001**, *31*, 38-42.
6. Khanna, S.; Venojarvi, M.; Roy, S.; Sharma, N.; Trikha, P.; Bagchi, D.; Bagchi, M.; Sen, C.K. Dermal wound healing properties of redox-active grape seed proanthocyanidins. *Free Radic Biol Med.* **2002**, *33*, 1089-1096.
7. Esposito, D.; Overall, J.; Grace, M.H.; Komarnytsky, S.; Lila, M.A. Alaskan Berry Extracts Promote Dermal Wound Repair through Modulation of Bioenergetics and Integrin Signaling. *Front Pharmacol* **2019**, *10*, 1058.
8. Vilkickyte, G.; Zilius, M.; Petrikaite, V.; Raudone, L. Proanthocyanidins from *Vaccinium Vitis-Idaea* L. Leaves: Perspectives in Wound Healing and Designing for Topical Delivery. *Plants* **2022**, *11*, 2615.
9. Kim, B.J.; Park, J.K.; Kim, B.K.; Park, S.J.; Kim, M.K.; Lee, C. won; Choi, L.M.; Hur, J.A.; Kim, S.H.; Beom, J.; et al. Oligomeric Procyanidins (OPCs) Inhibit Procollagen Type I Secretion of Fibroblasts. *Tissue Eng Regen Med* **2017**, *14*, 297–306.
10. Furumoto, T.; Ozawa, N.; Inami, Y.; Toyoshima, M.; Fujita, K.; Zaiki, K.; Sahara, S.; Akita, M.; Kitamura, K.; Nakaoji, K.; et al. *Mallotus Philippinensis* Bark Extracts Promote Preferential Migration of Mesenchymal Stem Cells and Improve Wound Healing in Mice. *Phytomedicine* **2014**, *21*, 247–253.
11. Fujita, K.; Kuge, K.; Ozawa, N.; Sahara, S.; Zaiki, K.; Nakaoji, K.; Hamada, K.; Takenaka, Y.; Tanahashi, T.; Tamai, K.; et al. Cinnamtannin B-1 Promotes Migration of Mesenchymal Stem Cells and Accelerates Wound Healing in Mice. *PLoS One* **2015**, *10*, e0144166.
12. Orlowski, P.; Zmigrodzka, M.; Tomaszewska, E.; Ranoszek-Soliwoda, K.; Pajak, B.; Slonska, A.; Cymerys, J.; Celichowski, G.; Grobelny, J.; Krzyzowska, M. Polyphenol-Conjugated Bimetallic Au@AgNPS for Improved Wound Healing. *Int J Nanomedicine* **2020**, *15*, 4969–4990.
13. Liu, K.; Zhang, C.; Chang, R.; He, Y.; Guan, F.; Yao, M. Ultra-Stretchable, Tissue-Adhesive, Shape-Adaptive, Self-Healing, on-Demand Removable Hydrogel Dressings with Multiple Functions for Infected Wound Healing in Regions of High Mobility. *Acta Biomater* **2023**, *166*, 224–240.
14. Pinto, S.C.G.; Bueno, F.G.; Panizzon, G.P.; Morais, G.; Dos Santos, P.V.P.; Baesso, M.L.; De Souza Leite-Mello, E.V.; De Mello, J.C.P. *Stryphnodendron Adstringens*: Clarifying Wound Healing in Streptozotocin-Induced Diabetic Rats. *Planta Med* **2015**, *81*, 1090–1096.
15. Zhao, Y.; Xie, Y.; Li, X.; Song, J.; Guo, M.; Xian, D.; Zhong, J. The Protective Effect of Proanthocyanidins on the Psoriasis-like Cell Models via PI3K/AKT and HO-1. *Redox Report* **2022**, *27*, 200–211.
16. Toda, K.; Tsukayama, I.; Nagasaki, Y.; Konoike, Y.; Tamenobu, A.; Ganeko, N.; Ito, H.; Kawakami, Y.; Takahashi, Y.; Miki, Y.; et al. Red-Kerneled Rice Proanthocyanidin Inhibits Arachidonate 5-Lipoxygenase and Decreases Psoriasis-like Skin Inflammation. *Arch Biochem Biophys* **2020**, *689*, 108307.
17. Tatsuno, T.; Jinno, M.; Arima, Y.; Kawabata, T.; Hasegawa, T.; Yahagi, N.; Takano, F.; Ohta, T. Anti-Inflammatory and Anti-Melanogenic Proanthocyanidin Oligomers from Peanut Skin. *Biol Pharm Bull.* **2012**; Vol. 35, 909-916.
18. Park, K.H.; Choi, S.E.; Choi, Y.W.; Lee, D.I.; Joo, S.S.; Jeong, M.S.; Bang, H.; Lee, C.S.; Lee, M.K.; Seo, S.J.; et al. Topical Application of Two Condensed Tannins from the Root of *Rosa Multiflora* Thunberg for the Treatment of Atopic Dermatitis (AD) in NC/Nga Mice. *Phytotherapy Research* **2011**, *25*, 1564–1569.
19. Shi, X.; Shang, F.; Zhang, Y.; Wang, R.; Jia, Y.; Li, K. Persimmon Oligomeric Proanthocyanidins Alleviate Ultraviolet B-Induced Skin Damage by Regulating Oxidative Stress and Inflammatory Responses. *Free Radic Res* **2020**, *54*, 765–776.
20. Chen, Y.; Huang, F.; McClements, D.J.; Xie, B.; Sun, Z.; Deng, Q. Oligomeric Procyanidin Nanoliposomes Prevent Melanogenesis and UV Radiation-Induced Skin Epithelial Cell (HFF-1) Damage. *Molecules* **2020**, *25*, 1458.

21. Matito, C.; Agell, N.; Sanchez-Tena, S.; Torres, J.L.; Cascante, M. Protective Effect of Structurally Diverse Grape Procyanidin Fractions against UV-Induced Cell Damage and Death. *J Agric Food Chem* **2011**, *59*, 4489–4495.
